# Real-space collapse of a polariton condensate

**DOI:** 10.1038/ncomms9993

**Published:** 2015-12-04

**Authors:** L. Dominici, M. Petrov, M. Matuszewski, D. Ballarini, M. De Giorgi, D. Colas, E. Cancellieri, B. Silva Fernández, A. Bramati, G. Gigli, A. Kavokin, F. Laussy, D. Sanvitto

**Affiliations:** 1 CNR NANOTEC—Istituto di Nanotecnologia, Via Monteroni, 73100 Lecce, Italy; 2 Spin Optics Laboratory, Saint Petersburg State University, 198504 St Petersburg, Russia; 3 Institute of Physics, Polish Academy of Sciences, Al. Lotnikow 32/46, 02-668 Warsaw, Poland; 4 Física Teorica de la Materia Condensada, UAM, 28049 Madrid, Spain; 5 Department of Physics and Astronomy, University of Sheffield, Sheffield S3 7RH, UK; 6 Laboratoire Kastler Brossel, UPMC-Paris 6, ÉNS et CNRS, 75005 Paris, France; 7 Università del Salento, Dipartimento di Matematica e Fisica “Ennio de Giorgi”, Via Arnesano, 73100 Lecce, Italy; 8 CNR-SPIN, Tor Vergata, viale del Politecnico 1, 00133 Rome, Italy; 9 Physics and Astronomy, University of Southampton, Highfield, Southampton SO171BJ, UK; 10 Russian Quantum Center, Moscow Region, 143025 Skolkovo, Russia

## Abstract

Microcavity polaritons are two-dimensional bosonic fluids with strong nonlinearities, composed of coupled photonic and electronic excitations. In their condensed form, they display quantum hydrodynamic features similar to atomic Bose–Einstein condensates, such as long-range coherence, superfluidity and quantized vorticity. Here we report the unique phenomenology that is observed when a pulse of light impacts the polariton vacuum: the fluid which is suddenly created does not splash but instead coheres into a very bright spot. The real-space collapse into a sharp peak is at odd with the repulsive interactions of polaritons and their positive mass, suggesting that an unconventional mechanism is at play. Our modelling devises a possible explanation in the self-trapping due to a local heating of the crystal lattice, that can be described as a collective polaron formed by a polariton condensate. These observations hint at the polariton fluid dynamics in conditions of extreme intensities and ultrafast times.

## Abstract (web summary)

Microcavity polaritons are a fluid of coupled photonic and electronic excitations that share many of the properties of Bose–Einstein condensates. Here, the authors show that the sudden creation of these bosonic fluids at high density results in the concentration of the particles, unlike an atomic gas that would expand.

## Graphical abstract

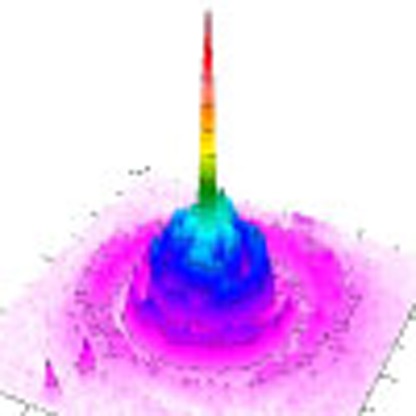

Microcavity (MC) polaritons^1^ have been praised for their fast response times^2^,^3^ and ease of manipulation as well as detection, inherited from the photonic component, while keeping a strong nonlinear character^4^, conferred by the excitons^5^. This makes them increasingly strong contenders in the field of interacting quantum fluids, where they have demonstrated the prevailing phases of strongly correlated systems^6^, including Bose–Einstein condensation^7^, superfluidity^8^ and quantized vortices^9^,^10^, together with rich spinorial patterns^11^ and nonlinear interference effects. Polaritons have also demonstrated their suitability to investigate another mainstream concept fuelled by dispersive and dissipative nonlinearities: shock waves and solitons, respectively characterized by step disturbances moving in the medium with sound velocity and by self-localization in space or shape preservation in time. Beyond polariton fluids^2^,^12^,^13^,^14^,^15^,^16^, these have also drawn much attention in nonlinear media^17^,^18^, atomic Bose–Einstein condensates (BECs)^19^,^20^,^21^,^22^,^23^,^24^,^25^,^26^ or microcavities in general^27^,^28^. For instance, the response of a nonlinear medium or atomic BECs to an impinging blast resulting in the irradiation of shock waves^20^,^29^, or the appearance of solitonic states^2^, have been recently reported. The time-resolved dynamics of such effects in strongly correlated gases remains largely unexplored and with the ultrafast imaging techniques now available, one may pursue a deep investigation of quantum fluid motion.

While they have reproduced most of the known phenomenology of quantum gases, polaritons also come with peculiarities of their own, such as their dispersion relation or their short lifetime, making them intrinsically out-of-equilibrium. Note that, in accordance with a widely spread terminology, we shall use the term ‘condensate' in our out-of-equilibrium context to refer to the coherent and macroscopically occupied polariton wavefunction, regardless of issues of spontaneous symmetry breaking, phase transitions and other important issues in a thermodynamic understanding of this term.

Here we report what appears to be a unique phenomenology of these systems, observed after the sudden coherent generation of a polariton condensate. The fluid undergoes a space redistribution leading to a central localization of a great number of polaritons, despite their repulsive interactions. The peak that is formed reaches a localization (≤2 μm, resolution limited) at least 10 times sharper than the initial Gaussian spot injected by the laser (18.5 μm). It also gathers a large number of particles, with a local enhancement up to 10 times the original density of polaritons. This striking dynamics takes place in a few ps. The enhancement factor and rise time can be tuned continuously with the excitation power. Another interesting feature of the dynamics is the generation of a shock wave at early times and concentric rings at later times. Similar rings and shock waves have been observed in nonlinear defocusing optical media or repulsive atomic BECs^18^,^24^,^29^, but the presence of a central localised and enhanced peak has never been reported so far in any system.

## Results

### Bright peak and centre collapse

To create a polariton fluid we used a typical MC sample containing three quantum well (QW) in the maxima of the electromagnetic field, embedded between two high reflectance multilayers mirrors (distributed Bragg reflectors)^4^,^8^. The strong coupling between the QW excitons and MC photons manifests itself as an anticrossing of their original modes splitting into two new normal modes, known as upper (UPB) and lower (LPB) polariton branches (see Supplementary Fig. 1).

**Figure 1:**
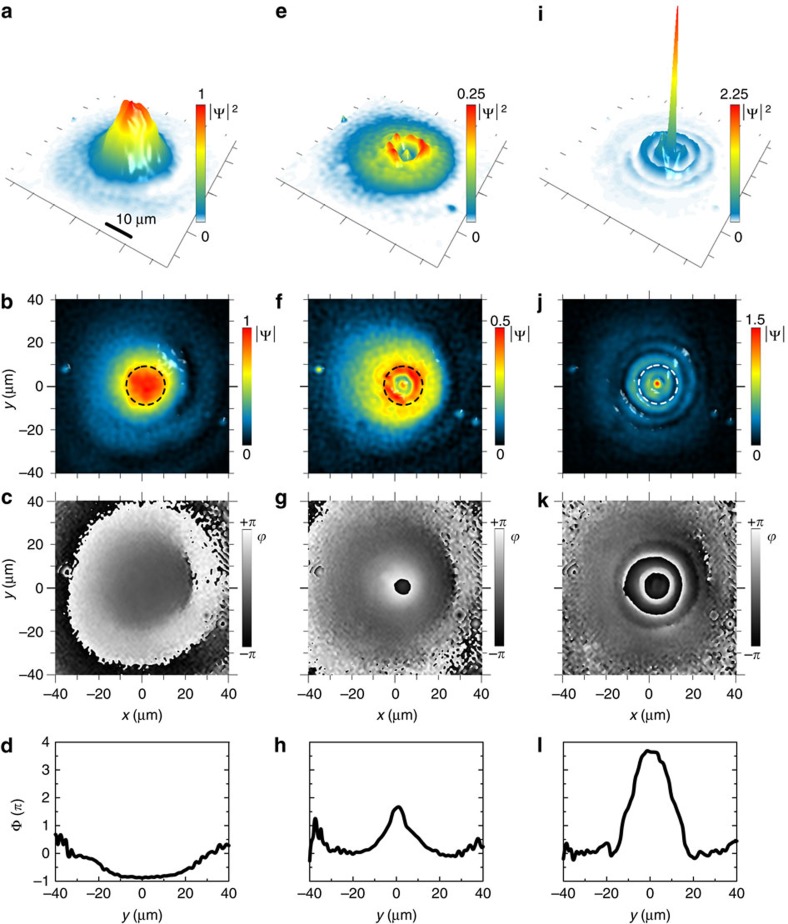
Snapshots of the polariton fluid density and phase at significant instants in the fs experiment. (**a**,**e**,**i**) (first row) Density maps of the planar polariton fluid on a 80 × 80 μm area as three-dimensional view and (**b**,**f**,**j**) (second row) amplitude maps as two-dimensional view (the dashed circles depict the initial pump spot FWHM). The three columns represent time frames at *t*=0 ps (**a**–**d**), 2.8 ps (**e**–**h**) and 10.4 ps (**i**–**l**). These time frames correspond, respectively, to the pulse arrival, the ignition of the dynamical peak and its long-lived state sitting at the centre of a ring structure (see also Supplementary Movies 1–3). (**c**,**g**,**k**) (third row) phase maps and (**d**,**h**,**l**) (fourth row) unwrapped phase profiles along the radius. The phase gradient subtends the superflow and here exhibits a reversal of the phase curvature, leading to the development of an opposite flow, toward the centre. The total number of particles intially excited in the whole area is 250 × 10^3^ polaritons (see Methods). The behaviour in time of the total population and of the centre density are shown in the Supplementary Fig. 2.

The dynamics of the polariton fluid, generated instantaneously by a resonant femtosecond laser pulse hitting the sample, is shown in Fig. 1. Initially, the polariton distribution is simply a footprint of the incoming laser spot, that is, a Gaussian of 18.5 μm full width at half maximum (FWHM). Panels (a–c) show the sample emission at the pulse arrival, in density 
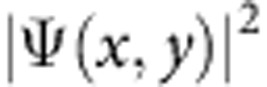
 (a), amplitude 
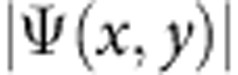
 (b) and phase 
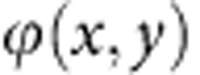
 (c). The unwrapped phase Φ(*y*) along a central diameter is shown in panel (d), with a weak (mean) outward gradient of 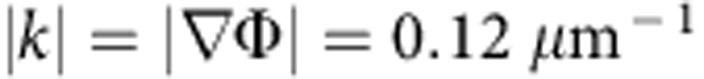, in the external regions. The central density of polaritons is initially 550 μm^−2^. For the first few ps, the total number of particles in the fluid only slightly decreases—due to radiative losses—however, at ∼2 ps, the centre density suffers a sudden depletion, and the maximum intensity is localized in a surrounding ring with only a quarter of the initial top density. This is shown in panels (e,f) for the intensity and (g,h) for the phase. This unanticipated jolt marks the beginning of the redistribution of the fluid. Note that the phase space profile starts to manifest a negative curvature with a reversal point of the phase gradient (∇Φ) at *r*=20 μm. Such an inversion in the phase gradient is an indication of a change in the fluid direction, from waves expanding outwards, to contracting towards the centre. This behaviour is then followed by the appearance of the bright and sharp central peak, contoured by concentric rings (i,j). This central peak collects 6% of the total particles still present in the fluid, and is surprisingly brighter than the initial spot was in the same area, despite the total population having largely decreased (to <1/3 after 10 ps). The behaviour of the total population and of the local centre density versus time can be seen in the Supplementary Fig. 2, highlighting an enhancement factor of 1.5 here, up to 10 times in other realizations (Supplementary Figs 3–5). Its localization goes below the resolution of our experimental setup and is thus <2 μm in width. The peak is furthermore robust as it occurs over the whole sample area, at all the different MC/QW detunings we have access thanks to the MC wedge (for example, between ∼0 and −1 meV, Supplementary Fig. 3 for a −0.8 meV negative detuning). It even sustains motion, as it actually propagates if imparted with an initial momentum (Supplementary Fig. 6). It is observed in the time-integrated camera images of the direct emission before any subsequent digital elaboration, which excludes any artifact of the technique (see also Supplementary Fig. 3, left column).

### Dynamical build-up of radial flows

**Figure 2:**
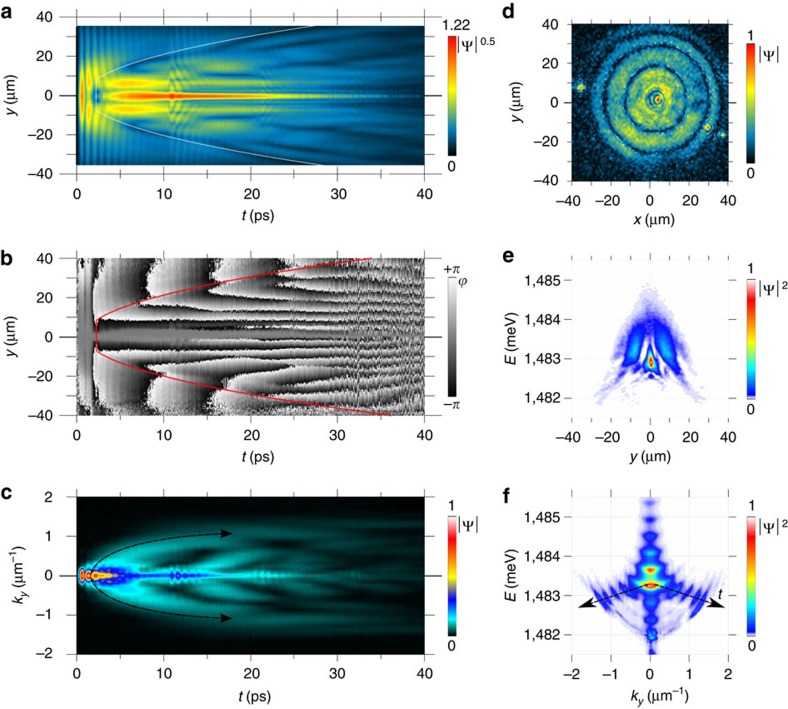
Dynamical charts of the complex wavefunction after fs excitation. (**a**) Time-space chart of the polariton amplitude 
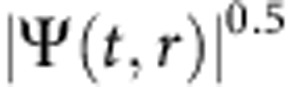
 (the square root is used to enhance the contrast) sampled with a timestep δ*t*=50 fs. The polariton fluid oscillates with a Rabi period of about 800 fs (vertical stripes in the map), while the central density rapidly decays to zero before starting to rise as a bright peak. An echo pulse due to a reflection from the substrate edge is visible at *t*=11 ps. (**b**) Time-space chart of the phase *ϕ*(*t*, *y*). In **a**,**b** two solid lines mark the phase disturbance delimiting the expanding region with large ∇Φ. (**c**) The time evolution of amplitude in momentum space, 
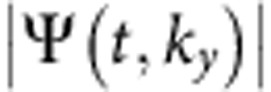
. The initial polariton population, featuring a very narrow Δ*k* width (imparted by the photon packet), ejects an expanding disk developing into a ring. (**d**) 
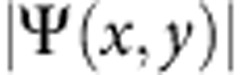
 map at *t*=26 ps, showing the dark/bright ring structures. (**e**) A *y*−*E* cut showing the energy of the fluid along the diameter. The central brightest spot is less blueshifted than its sides. (**f**) Time-integrated *E*−*k*_*y*_ dispersion under the femtosecond coherent excitation. The dashed arrows depict the opening up in the *k* space and are associated to the dashed lines in **c**. The periodic oscillations in the energy domain of **f** are due to interferences of time-delayed reflections from the substrate edge as explained in the Methods section.

The full dynamics of the polariton fluid and the connection between the density accumulation and the radial flows are further studied in Fig. 2, yet again in the case of fs pulse excitation. Here are shown the amplitude and phase profiles versus time (panels (a) and (b)). The amplitude chart reveals the bright peak as a central horizontal line which reaches its maximum intensity at a time of ∼11 ps. The vertical stripes observed in the first 5–6 ps are Rabi oscillations^3^,^30^ between the excitonic and photonic fields. The period of *T*_R_∼800 fs corresponds to the energy separation between the UPB and LPB of 5.4 meV, while their fast decay is due to the rapid scattering of the UPB polaritons. The Rabi oscillations are triggered by the femtosecond pulse that excites simultaneously both polariton branches (9 nm energy width, Supplementary Figs 1,2). They do not, however, play an important role in the observed phenomenology, since the dynamical localization also happens under the excitation of the LPB alone by a picosecond laser pulse (0.35 nm width, Supplementary Fig. 7). This confirms the robustness of this peak. Since it has an homogeneous phase, as can be seen from the phase graph in Fig. 2b, it is a standing wave. We also note that a neat time-space cone marks a boundary between two regions: an expanding internal domain with almost horizontal black and white (b/w) bands (strong inward ∇Φ) and an external domain with almost vertical b/w bands (null or weak ∇Φ). This is a clear evidence that a circular front of phase disturbance (marked also by a low density, solid lines in panels a,b) is expanding, leaving after its passage a structure of multiple rings, as illustrated in the still image of panel (d). If an attractive term is at play here, it thus seems to be in the course of expanding its range of action.

The observed phase disturbance seems to come out from a central region (at *r*=half width at half maximum (HWHM)) with a speed of 3 μm ps^−1^ and then decreases to an almost constant cone angle representing a velocity of 1 μm ps^−1^ at later times. The speed of sound is related to the density of the fluid according to the well known equation 
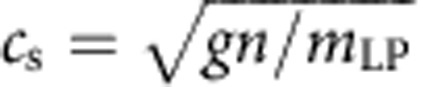
, with *g* the nonlinearity, *n* the density (or particles number) and *m*_LP_ the effective mass of the lower polaritons. Using the equation for the measures of Fig. 2, it gives a *c*_s_=1.5 μm ps^−1^ (centre density) and 1.1 μm ps^−1^ (at the HWHM radius), at the initial time. Hence, the phase disturbance is at least two times faster than the speed of sound initially, decelerating then as long as it propagates outside (apparently by a factor of 
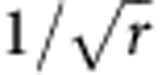
). Since also density decreases both along the distance and with time, the phase disturbance behaves as a shock wave which always keeps supersonic.

To further characterize the flow dynamics and its role in the formation of the central peak, we extend our study to the reciprocal space, reporting the one-dimensional cross section of the *k*_*x*_, *k*_*y*_ plane in panel (c) of Fig. 2. The initial width Δ*k* of the polariton population created by the laser pulse is very small (∼0.24 μm^−1^ FWHM) and concentrated around *k*=0. After a couple of picoseconds, the fluid suddenly ejects a disk in *k* space which stabilizes within 10 ps into a ring at finite momenta around 
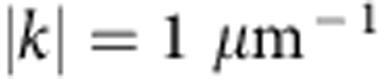
, associated to the inner growing flow. These values correspond to a *v*=1.6 μm ps^−1^ velocity, using the formula for the fluid velocity *v*=*ħk*/*m*_LP_ (holding in the parabolic approximation, valid up to the inflection point at 1.5 μm^−1^). Hence, also the radial flows created inside the boundary regions are supersonic. Looking at the onset of the Rabi oscillations allows us to make a correspondence between real and reciprocal space, indicating that the central bright peak in real space is associated to the finite momenta travelling waves in reciprocal space. The energy of these waves is shown in the integrated dispersion of Fig. 2f. Note that, as oberved in the dispersion plotted in Fig. 2f, the ring in *k* space gradually expands from *k*=0 at early times (maximum blueshift of the polariton population) to the value of 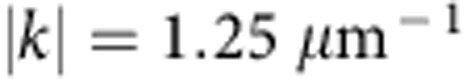 when the dispersion is redshifted to its original bare energy. The space-energy profile in Fig. 2e explains in part the inner flow. Here it is clear that the central part of the spot is redshifted with respect to the sides, hinting at the presence of an effective attractive potential responsible for the central peak of high-polariton density. Although it is not clear at the outset what the origin of such a potential is, it is consistent with an attractive term (nonlinearity inversion) or with nonlocal interactions, that is, *k*-dependent blueshift^31^,^32^. In our case, we may reasonably infer that the inward coherent waves generated by such a potential interfere with opposite *k*_*r*_ vectors, explaining the ring structure and the central accumulation enhanced by a polar scaling of 1/*r* which is typical of an interfering ring wave (Supplementary Fig. 8).

### Dark rings

**Figure 3:**
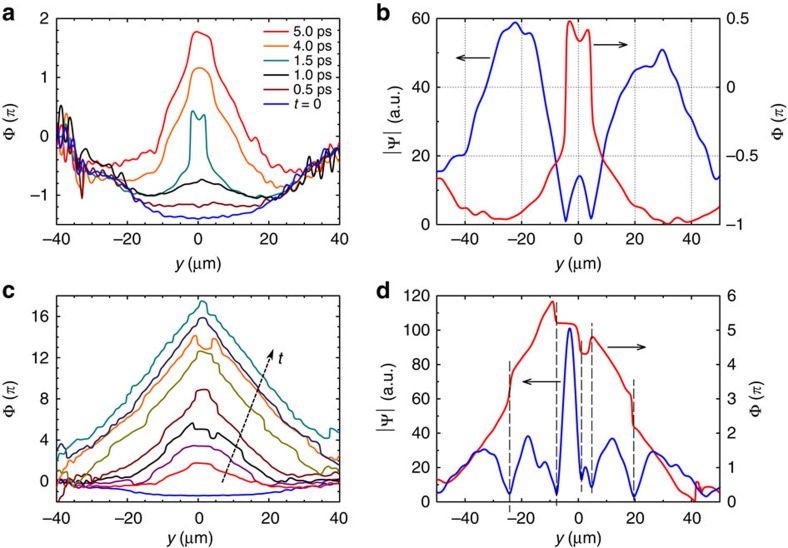
Phase crosscuts during the fluid evolution and signature of dark ring solitons. (**a**) Unwrapped radial phase profile at early time, showing the reversal of the phase curvature. (**b**) The sudden phase switch at *t*=1.5 ps is shown together with the associated amplitude profile. The dip in the intensity with the *π*-jump in phase is a signature of radial interference and of a possible dark ring soliton, surrounding the bright peak. (**c**) Radial phase profile at later time, taken each 5 ps over a 0–40 ps timespan. The phase slope increases with time up to ∇Φ∼2*π*/(5 μm)≃1.25 μm^−1^. (**d**) Amplitude and phase profiles at 16 ps showing that a nonlinear interference is reshaping the fluid in a series of concentric rings.

To verify the nature of the interference, we report the phase and amplitude profiles of the fs experiments at different time stills. In Fig. 3a, the unwrapped phase profile during the first 5 ps is shown. Soon after the phase reversal, a sudden, almost instantaneous switch happens at *t*=1.5 ps. The rigid switch is a *π*-jump, corresponding to the dark notch in the density, as seen in panel (b). This is a typical sign of an interference between counterpropagating waves. In the following dynamics, as already said, the reversed gradient tends to grow and expand all over the originally excited area, as depicted in panel (c) with profiles at 5 ps intervals. Other *π*-jumps in the phase can be observed at a later time, corresponding to dark rings in the density, see Fig. 3d. This is an evidence that interference phenomena of coherent waves are acting in reshaping the fluid density as a series of concentric rings. It also suggests a possible link to—without being a ‘per-se' proof of—ring dark solitons^33^. These specific solutions to nonlinear Schrödinger equations (NLSE) under repulsive interactions are predicted to become stable in the case of two-dimensional fluids, such as polaritons^34^. It appears that a ring dark soliton indeed holds for the first dark ring around the rising bright peak, given its stability for several tens of ps.

### Nonlinear drive

**Figure 4:**
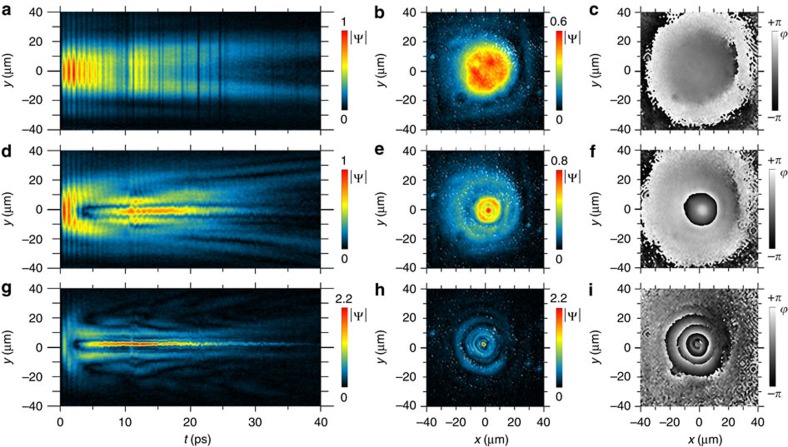
Time-space charts and space maps for different femtosecond pulse power. (**a**,**d**,**g**) Time evolution of the radial modulus 
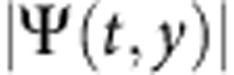
 for three different powers (left column), (**b**,**e**,**h**) relative amplitude 
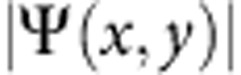
 and (**c**,**f**,**i**) phase *ϕ*(*x*, *y*) maps at *t*=12 ps (mid and right columns, respectively). Increasing the initial density leads to a faster central depletion and stronger rise-back reaction. In the third row the dominating feature is the bright peak, with an enhancement factor of almost 5 in intensity, while outradiated waves are faster but almost cancelled out on a relative scale. This demonstrates the strong nonlinearities acting in the central gathering of polaritons and in setting the radial momentum and speed of the ring waves. The three rows refer to initial total populations (initial top density) of 25 × 10^3^ (55 μm^−2^), 125 × 10^3^ (275 μm^−2^) and 450 × 10^3^ polaritons (1,000 μm^−2^), respectively, excited by femtosecond pulses.

The nonlinear nature of the effect is demonstrated in Fig. 4, relative to femtosecond excitation with different pulse powers. At low density, Fig. 4a–c, the polariton condensate behaves as expected from any fluid freely released, with a small diffusion and remaining homogeneous in both density and phase, as well as, in our case, Rabi oscillations at earlier times. It is also clear that the expansion speed imparted by the small initial Δ*k* is negligible. At five times higher excitation power (Fig. 4d–f), there is a density redistribution of polaritons to form the central localization peak, surrounded by ring structures and out-radiating shock waves, however, yet without a strong enhancement and a moderate phase reversal. In Fig. 4g–i, at 18 times the initial pumping power, the structure gets fully formed, with a central peak gathering over twice the population locally present at the initial time and with a much steeper phase reversal, giving rise to the striking structure in Fig. 4h. Here again we emphasize that the central peak is resolution limited and is likely sharper than is resolved in our experiment. Additional examples with fs excitation are provided in the Supplementary Fig. 3 for an extended set of excitation densities.

## Discussion

We now discuss which physical mechanism could be responsible for such a remarkable phenomenology. The strong-coupling regime of light and matter at the core of the polariton physics can lead to distinctive dispersive and dissipative nonlinearities^5^,^35^,^36^,^37^. For instance, polaritons support dissipative solitons^38^, have demonstrated bistability domains with a switching on/off of both bright and dark solitons^39^ as well as moving bright solitons along a steadily pumped background^1^,^2^,^14^ (this last based on the negative curvature of the polariton dispersion above the inflexion point). All these features are accountable by one of the several models used to describe polariton fluids. While the positive nonlinearities intrinsic to polariton interactions, due to the excitonic repulsions, are supposedly able to force the expansion and reshaping of a polariton fluid and to sustain dark solitons^33^, possibly shedding light to some aspect of our experiment, there is no documented mechanism to explain the most striking feature: the real-space collapse in the centre of the spot. Real-space localization could in principle appear under negative, that is, attractive nonlinearities^5^. As we review below all the obvious candidates to account for the observed phenomenology, we can rule out this and other tempting explanations (see also Supplementary Notes 1–4 and Supplementary Figs 9–13). Our analysis will show that the most likely origin of the nonlinear activation of a central bright spot is the self-trapping of a polariton condensate by a type of collective polaron effect (see Supplementary Note 5 and Supplementary Figs 14–19).

A first possibility to explain the self-localization is for the polariton population at early times to undergo a transition to the weak coupling regime (screening and reduction of coupling). Although this can be partially happening at the largest power (given the excited density there is 0.33 × 10^3^ μm^−2^ per QW and approaches values of the Mott density, in the order of 2–5 × 10^3^ μm^−2^), it does not explain the effect since (i) the blueshift of the LPB is continuous and does not reach the photonic mode; (ii) increasing power/density does not increase the size of the bright peak, as expected if merely enlarging the size of an above-threshold region; (iii) the total intensity decays with the LP polariton lifetime of 10 ps (Supplementary Fig. 2) and (iv) transition to weak coupling for excitation below the band edge would manifest as a higher blueshift as follows from the nonlinear Kramers–Kronig relations^40^,^41^, while we observe a lesser blueshift in the centre with respect to the side bright ring, as shown in Fig. 2e. An exciton reservoir, separate from the polariton fluid, is known to play a significant role in many polariton experiments. We can exclude it in our case for the following reasons: (i) the effect persists when resonantly exciting the LPB, a configuration that does not populate the reservoir (see the Supplementary Fig. 7 on the picosecond experiment), (ii) a moving bright peak is observed when exciting with nonzero initial in-plane wavevector *k*, ruling out a reservoir that would need to be dragged by the structure as it propagates, which is impossible given the heavy mass of the reservoir excitons (see the Supplementary Fig. 6 on the moving peak), (iii) the effect does not show any strong polarization dependence. These considerations thus exclude the case of a dissipative bright soliton^38^ predicted under cw pumping where the localization is sculpted by an interplay between source and decay regions and the compensation of their steady flows. The appealing recourse to attractive interactions cannot be sustained either. While polariton attractions are possible due to the various superexchange processes through dark excitons or bi-exciton states, or due to Van der Waals forces, none of these mechanisms can account for the experiment in a careful analysis. The former mechanism should be strongly polarization dependent^5^,^35^, which is not our case, and the latter are too small with respect to the repulsive Coulomb or exchange terms^5^. Similarly, non-locality (*k*-dependence, which could also lead to a negative dispersion in the centre of the spot^31^) of one or more of these terms and even retardation effects are negligible as well^32^ and fail to produce the real-space collapse in numerical simulations. Intriguingly, the dynamical Casimir effect^42^ recently proposed^43^,^44^ in a configuration very similar to our experiments, was predicted to generate finite momentum excitations from the vacuum adding up to the suddenly excited fluid at *k*=0. However, this effect, was considered only for the one-dimensional case of polaritons at zero temperature, and the model is not ready to be compared at its stage of development with the configuration of our experiments (see the Supplementary Note 6).

**Figure 5:**
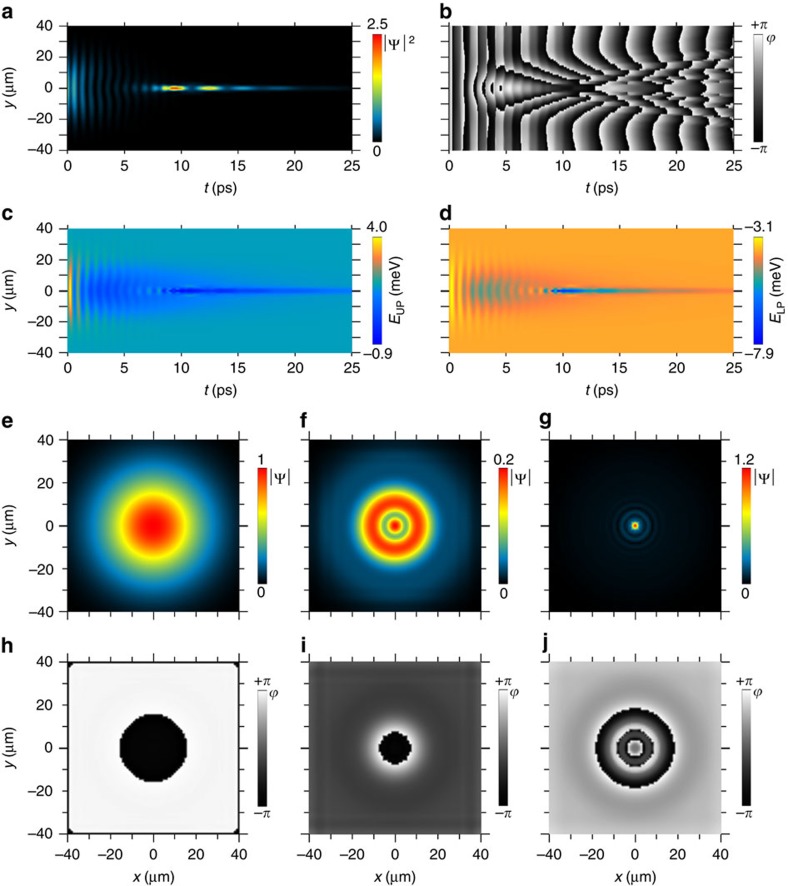
Theoretical time-space charts and *xy* maps of the polaron model. (**a**,**b**) The calculated magnitude of the polariton fluid as a function of *y*-coordinate and time (**a**) together with its phase (**b**). (**c**,**d**) The corresponding calculated energy profiles for the upper and lower polariton branches are shown in **c**,**d**, respectively. (**e**–**g**) Spatial maps of the wavefunction amplitude for the time *t*=0, 4 and 13 ps and associated (**h**–**j**) phase maps. The presented case correponds to the power *P*_6_ in the series of the Supplementary Figs 15–16.

The failures of these analyses point at an unconventional mechanism ruling the high density, ultrafast dynamics of polaritons (see also the Supplementary Discussion). Given that the object appears to be self-sustained, it is important to elucidate its nature, as it may have important applications, especially as the control of ultra-sharp localized light peaks is clearly of technological interest, for instance for high resolution displays or memory units. One of the unconventional scenarios that we found to be fairly consistent with most the hypotheses and observations of our experiments involves a type of collective polaron effect. The recent work by Klembt *et al*.^45^ shows that the resonant pumping of exciton polaritons into a MC may result both in cooling or heating of the crystal lattice depending on the initial lattice temperature and the optical pump power. In our experiments, realised at a cryogenic temperature and sufficiently high pump power, one should expect such a local heating of the crystal lattice due to the polariton Auger process, followed by the emission of a cascade of acoustic phonons. The probability of this process is quadratic in the polariton density. The heating results in the local band-gap renormalization which is responsible for the redshift of the exciton energy. The heating by 20–30 K results in a redshift of the exciton energy of 1–2 meV, which is sufficient for trapping the polariton fluid. In this way, a trap in real space is formed under the pump spot. It becomes deeper as more polaritons are getting trapped, thus providing a positive feedback that stabilizes the self-trapping process and explains the robustness of the effect. The proposed model would also explain the case of a moving bright soliton^1^,^2^, where a compensation of positive nonlinearity and negative dispersion above inflexion point, only justifies the localization along the propagating direction and not in the transverse direction, where just a positive parabolic dispersion applies. We have modelled the self-trapping through a generalized Gross–Pitaevskii equation, described in the Supplementary Note 5. Our collective polaron model yields the best fit to the data with a heat relaxation time *τ*_H_=8 ps, hence the fluid redistribution and the heat relaxation dynamics appear to be of similar timescales. We evaluated a velocity for the outward heat diffusion of 0.1 μm ps^−1^, lower than the polariton propagation velocity. Figure 5 shows a result of the numerical simulation for the wavefunction of the polariton fluid ruled by this process. The upper panels (a,b) show the real-space dynamics and next panels (c,d) the energies of the LPB and UPB, respectively. Beyond the dynamics of the Rabi oscillations, in particular their bending, the model also reproduces the self-localization in good qualitative agreement with the experimental data (see panels (e,f,g) and (h,i,j) for the real space maps of amplitude and phase, respectively, to be compared with the analogue experimental ones of Fig. 1). The enhancement factors (up to 2.5) achieved in the polaron model are comparable with the experimental ones, with some deviations at the larger pumping powers. The values of the rise time show a decreasing trend with the initial density (from 14 to 8 ps) in a very good agreement with the experiments (from 19 to 5 ps; Supplementary Fig. 17).

In conclusion, we have observed the dynamical appearance of a bright and sharp peak sitting at the centre of a series of concentric rings in a polariton fluid generated by the sudden excitation from a resonant laser pulse at *k*=0. The peak that appears at high pumping is robust to other variations in the experimental parameters (detuning, momentum, and so on), is resolution limited and gathers up to 10 times the population initially present in its area. This striking structure cannot be explained by any of the conventional mechanisms such as loss of strong-coupling nor by the common models of polariton dynamics, including Gross–Pitaevskii type of equations with or without reservoirs and/or attractive interactions. We have provided a possible intepretation in terms of the collective polaron effect, resulting in a self-trapping of the polariton fluid. Our results show that much is left to explore in the high-density and ultrafast dynamics of polaritons, with a unique phenomenology that could stimulate new areas of research and applications.

## Methods

### Experimental configuration

The polaritonic sample used here is an AlGaAs 2*λ* MC with three In_0.04_Ga_0.96_As QWs of 8 nm placed at the antinodes of the cavity mode field. The cavity is embedded between two distributed Bragg reflectors made of 21 and 24 pairs of alternated *λ*/4 AlAs and GaAs layers. The sample exhibits an excellent quality factor (photonic *Q*=12,000), resulting in a lower polariton lifetime *τ*_LP_=10.7 ps at zero detuning. All the experiments shown here in the main text are performed at zero detuning between the MC and the QW exciton, both resonant at ∼834.8 nm (1,485.2 meV), and in a region of the sample clean from defects in order to avoid any effect due to spatial inhomogeneities. The device is kept at a temperature of 10 K. We implemented an ultrafast imaging technique based on the off-axis digital holography^3^,^10^,^46^,^47^ to study the dynamics of the polariton flow with spatial and temporal steps of 0.16 μm and 50 fs, respectively. A 130 fs (9 nm bandwidth) or 3.5 ps (0.4 nm bandwidth) laser pulse, quasi-resonant with the bottom of the LPB and circularly or linearly polarized, is directed onto the sample at normal incidence. The central energy of the fs pulse does not play a major role if changed in a few nm range around resonance, apart from setting a different initial density of polaritons. The central energy of the ps pulse has to be set instead slightly higher (0.1–0.5 nm) than the LPB mode, in order to give an appreciable initial blueshift to the polariton fluid. It cannot be set further higher because the dynamical redshift of the polaritons would go out of the energy range of the reference pulse (which is a twin copy of the exciting one), preventing the interference at the basis of the detecting technique. The evolution of the polariton fluid in time is recorded by making interfere the sample emission with a delayed reference beam into a charge-coupled device (CCD) camera. Digital elaboration in the reciprocal space of the interferograms allows to retrieve the complex wavefunction of the photonic emission, which is coherent with the polaritonic wavefunction, and thus allows us to image both the amplitude and phase of the light-matter fluid. The snapshot images of the real space (*y*, *t*) or reciprocal space (*k*_*y*_, *t*) at a given time are obtained from interferograms integrated over thousands of pulses. The repetition rate of our pulsed laser is *f*=80 MHz, while the integration time *τ*_CCD_ used in the CCD camera is set in a range between 0.15 and 1.0 ms, meaning that we are integrating a number *N* of shots, with 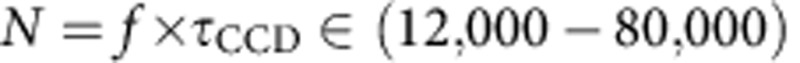. In our interferometric setup, the visibility of the fringes remains stable for *τ*_CCD_≤1.0 ms, which cuts out the mechanical vibrations of the setup. The total polariton populations were evaluated by measuring the time-integrated real space emission using a power metre (calibrated photodiode). In the pulsed regime, the emitted power is equal to the energy per emitted shot multiplied by the pulse train frequency. Hence we can evaluate the number of emitted photons per shot, which is equal to the initial total population of polaritons if assuming that all the polaritons initially present (at *t*=0) in the fluid are radiatively emitted due to the photonic losses (that is, neglecting other losses). The initial centre density (intensity) was evaluated by using the mathematical relations between the centre density in a Gaussian spot and its area-integrated intensity. The experiments were performed by using lenses with 10 and 3 cm focal length, on the excitation and detection sides, respectively. There is no selection in *k* space performed by the excitation scheme, and the excitation spot is Gaussian in both real and reciprocal space, with the widths respecting the Fourier relation, as clear from the data in Fig. 2a,c. For energy resolved images a standard 550 mm long spectrometer is used before the CCD and the spectra are time integrated. Please note that the apparent discretization in energy appearing in Fig. 2f is given by the interference in the energy domain of time-delayed reflections back from the substrate edge and is not affecting the ongoing dynamics. The substrate has got a 1.5-mm-optical thickness, associated to a 10 ps time distance of the reflected echos of the emission and to a 0.45 meV fringes spacing in energy. The emission echo is visible also in the time-space measurements of real and reciprocal space (Fig. 2a,c), where it is seen weaker and weaker at regular interval of 10 ps. Additional details on the sample and the technique can be found in ref. 3 and the supplemental material therein.

## Additional information

**How to cite this article:** Dominici, L. *et al*. Real-space collapse of a polariton condensate. *Nat. Commun.* 6:8993 doi: 10.1038/ncomms9993 (2015).

## Supplementary Material

Supplementary Information Supplementary Figures 1-19, Supplementary Notes 1-6, Supplementary Discussion and Supplementary References

Supplementary Movie 1 The 3D movie shows the complete imaging of the polariton fluid dynamics on a 40 x 40 μm2 area and over a timespan of 40 ps with a timestep of 50 fs. The representation of the photonic field with height proportional to the amplitude |Ψ(x,y)| at each time frame gives a direct view of the ultrafast superfluid dynamics, comprising initial Rabi oscillations and the central collapse and enhancement as described in the text and in Fig. 1-3.

Supplementary Movie 2 The 2D movie shows the complete imaging of the polariton fluid dynamics over a timespan of 40 ps with a timestep of 50 fs. The 2D representation of the amplitude |Ψ(x,y)| allows to appreciate the formation of a ring structure at later times, as the one shown in Fig. 1j and 2a.

Supplementary Movie 3 Same as the previous movies but now showing the corresponding radial cross section of the polariton density |Ψ(y)|2 at each time frame. As an interesting detail, it is possible to observe how the initial expansion in the first few ps is happening during the half-periods of the Rabi cycles where there is a low photonic density (i.e., a high excitonic one).
