# Supplementary material for: Real-space collapse of a polariton condensate
Source: Nat Commun. 2015 Dec 4;6:8993. doi: 10.1038/ncomms9993 (PMC4686858; doi:10.1038/ncomms9993)
Supplement: Supplementary Information — Supplementary Figures 1-19, Supplementary Notes 1-6, Supplementary Discussion and Supplementary References [file ncomms9993-s1.pdf]

SUPPLEMENTARY FIGURES

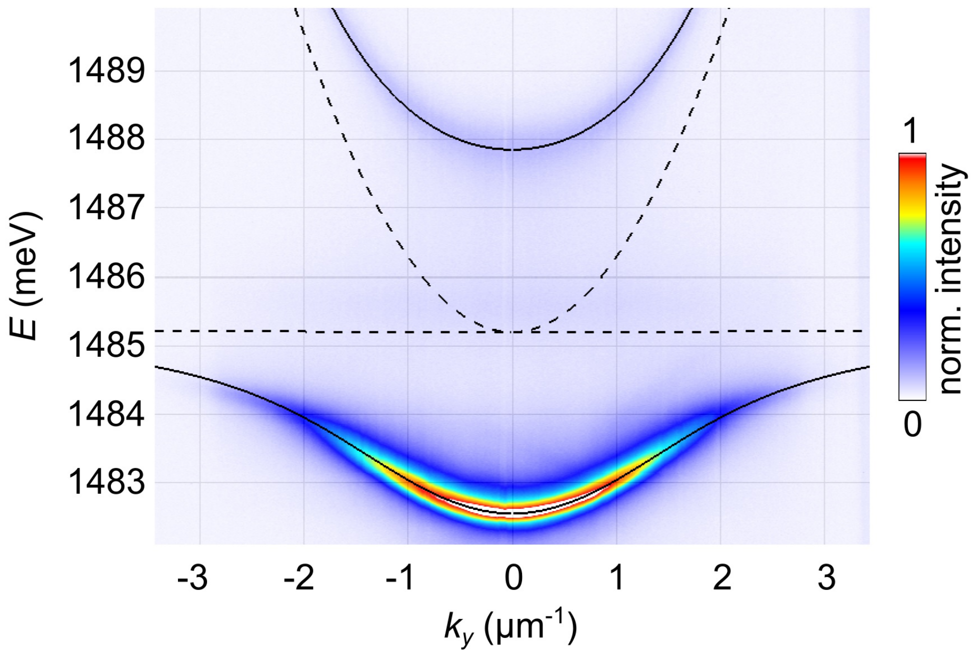

Supplementary Figure 1. Polariton dispersion. The bare E–k emission of the microcavity polaritons is obtained after cw off-resonant excitation at low power. The separation of the UPB and LPB branches is 5.4 meV (3 nm).

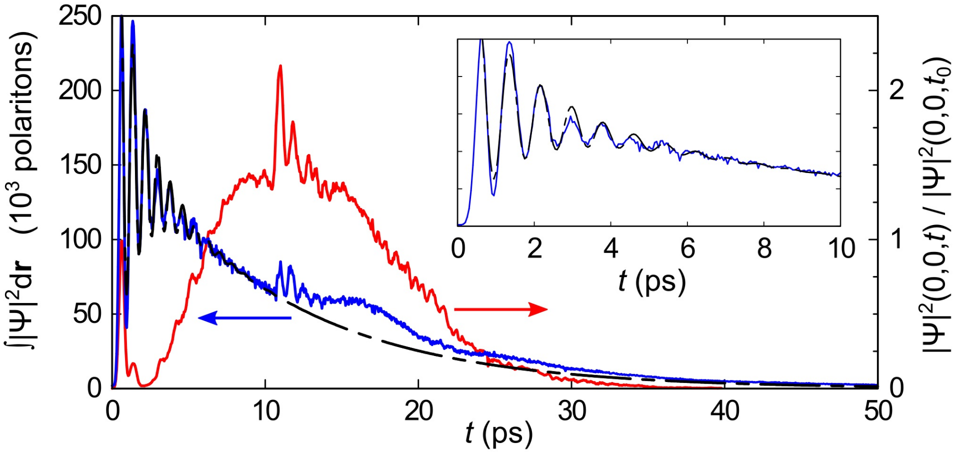

Supplementary Figure 2. Total population and density at the center versus time in the femtosecond experiment of Fig. 1-3 of the main text. Blue line are the experimental data of the area-integrated emission intensity sampled every 50 fs. The black line is a fit based on a model of coupled and damped oscillators, retrieving a lower polariton lifetime of $\tau_{\rm LP} = 10.7$ ps. The inset is an enlargement of the first 10 ps. The red curve to be plotted on the right axis is the centre density versus time relative to that at the time of pulse arrival ($t_0 = 0.6$ ps). The real enhancement factor obtained here in the centre density is 1.5, reached in a rise time of ~ 10 ps, while the effects of the emission reflection arriving at $t = 11$ ps and momentarily affecting (kicking it up) the centre density should not be taken into account.

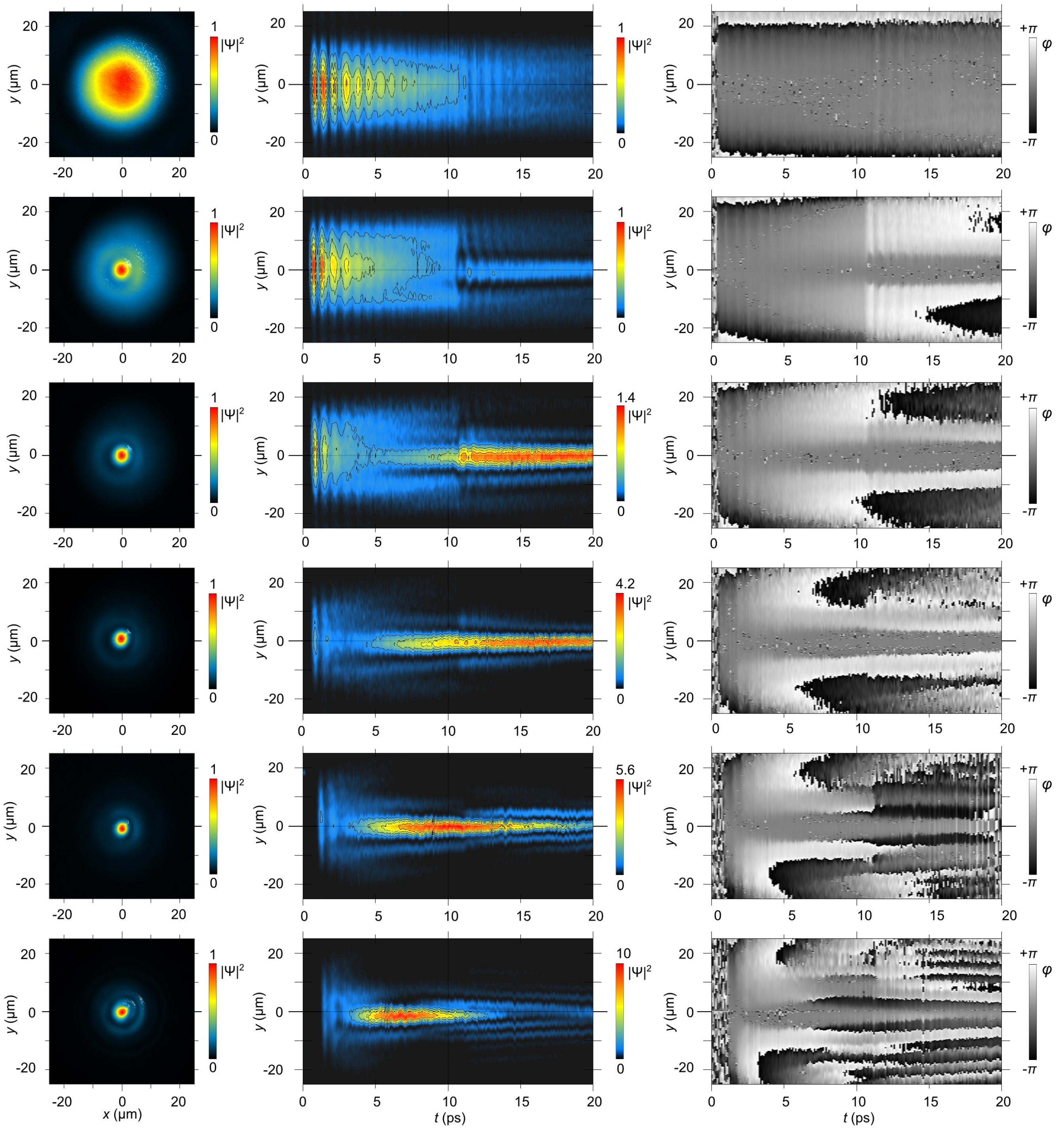

Supplementary Figure 3. Femtosecond experiment with a 16 µm FWHM gaussian spot and linear polarization in a position with a microcavity-exciton negative detuning (-0.8 meV). Each row is relative to a different initial density. The first column represents the time-integrated images of the bare emission in space directly aquired on the camera. The second and third columns represent the density and phase profiles, respectively, along a central diameter and versus time, retrieved by means of the ultrafast imaging. The powers are increasing from top to bottom rows and correspond to initial total populations of P_1–$P_6 = 20 \times 10^3$, 40×10^3, 70×10^3, 110×10^3, 200×10^3 and 420×10^3 polaritons. The associated initial top densities are: 70 µm^{-2}, 140 µm^{-2}, 240 µm^{-2}, 380 µm^{-2}, 690 µm^{-2} and 1440 µm^{-2}, respectively. The maxima on the colour bars of the density charts are expressed in terms of the initial top densities and represent the achieved enhancement factors.

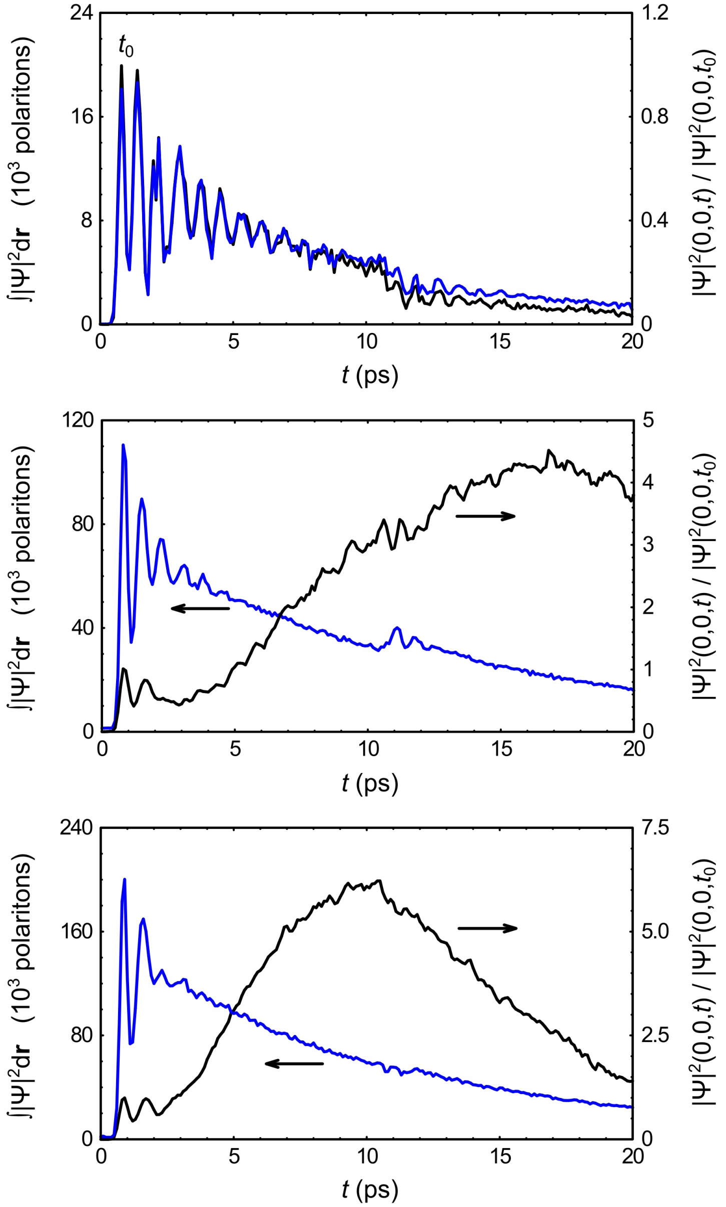

Supplementary Figure 4. Total density and density at the center in the case of the femtosecond experiments of the previous Supplementary Figure 3, for the excitation powers P_1, P_4 and P_5 as a function of time. The blue curve plotted on the left gives the total number of polaritons with time while the black curve plotted on the right gives the corresponding centre density as a ratio with respect to the one at the time of the first peak (t_0).

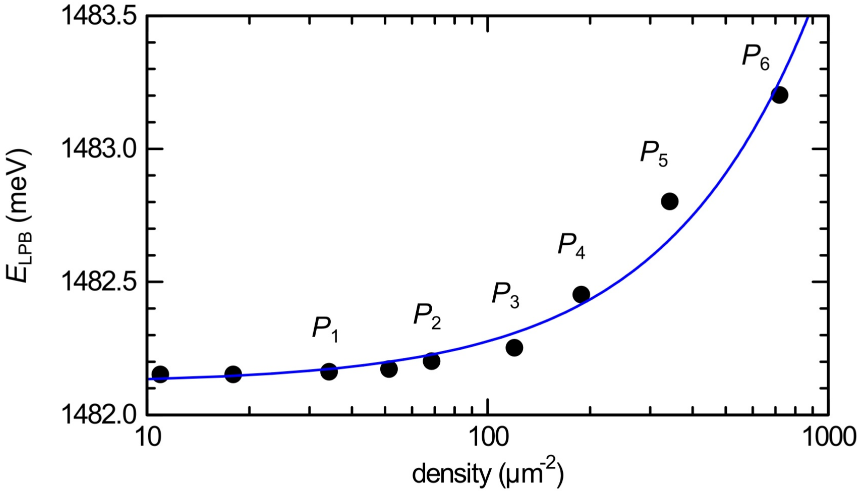

Supplementary Figure 5. Blueshift at different femtosecond excitation powers. The experimental series is the same as the previous Supplementary Figures 3 and 4, with labels indicating the P_{1-6} powers. The blueshifted values are taken from time-integrated far-field dispersion measurements. We report in the y-axis the blueshifted energy of the brightest point in the far-field measurements and in the x-axis the mean density values of the real-space 2D gaussian. The solid line is a linear fit to the data which gives a nonlinearity of $g \sim 1.5$ µeVµm^2. We assume that the total population is equally distributed between the three QWs and that each results in the same blueshift. Hence the overall nonlinearity should be renormalized to $g \sim 4.5$ µeVµm^2 when referring to the single QW.

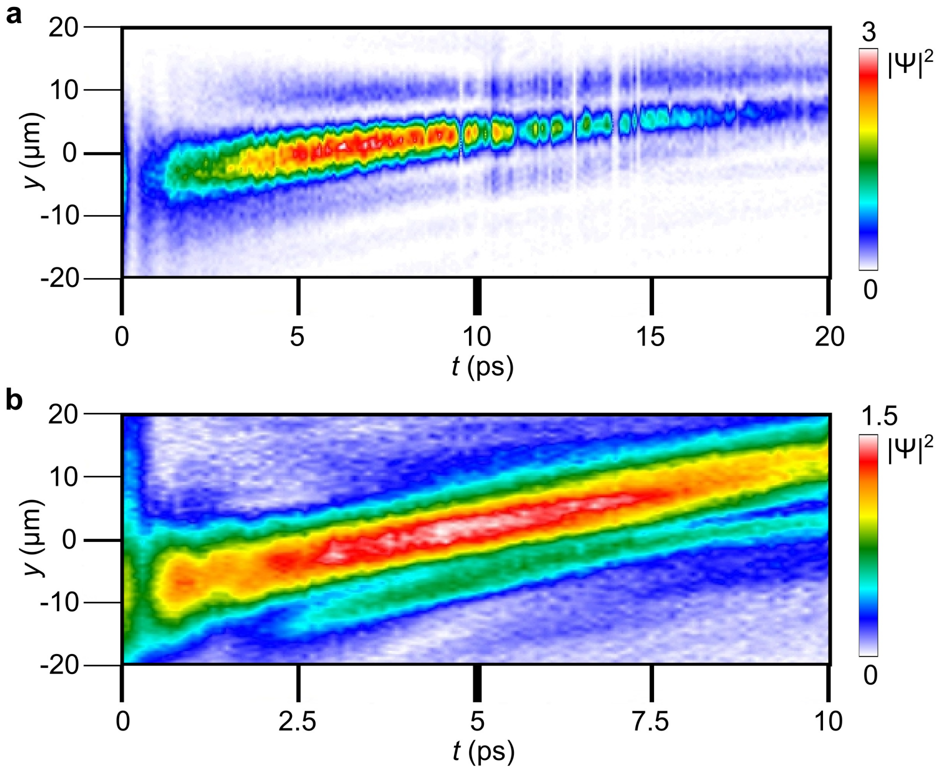

Supplementary Figure 6. Propagating bright peak activated under resonant fs pulse injection with (a) 0.7 µm^{-1} and (b) 1.6 µm^{-1} in-plane wavevectors k_y. The cuts of the spatial polariton density as a function of time are taken along the propagation direction. The colour bars represent the density relative to the top density at the initial time (in the present case $t_0 = 0$).

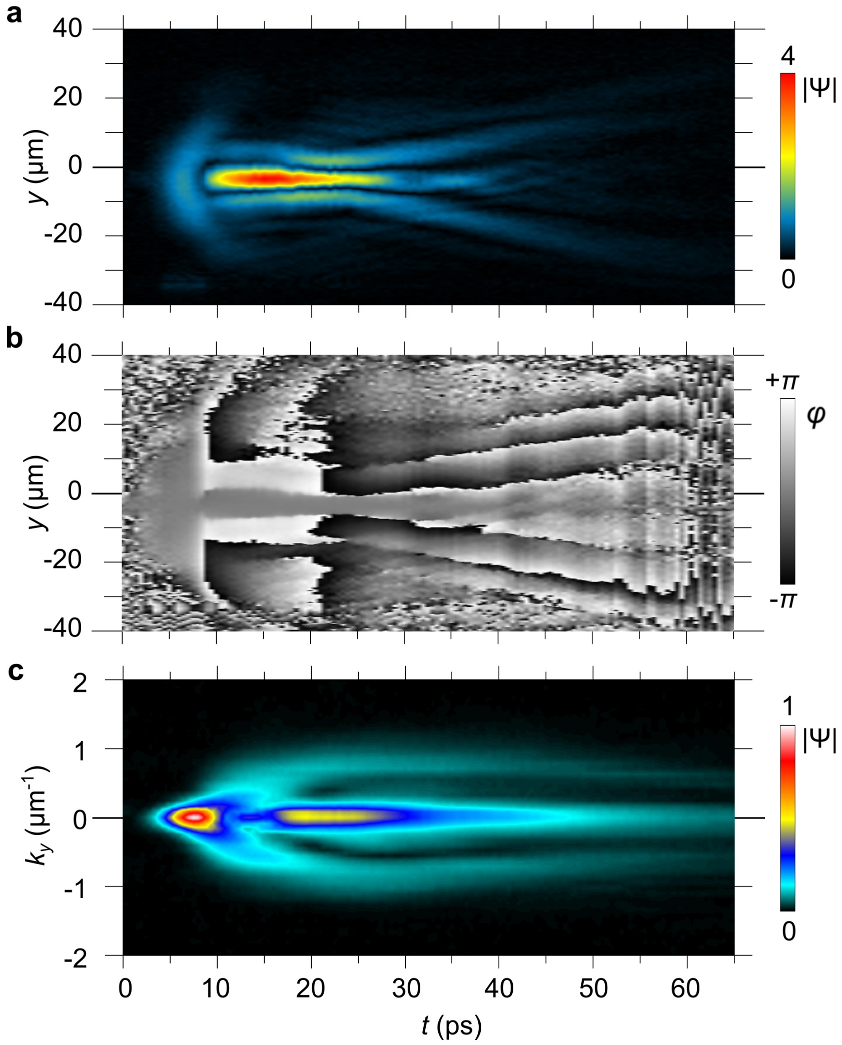

Supplementary Figure 7. Picosecond experiment. The excitation pulse is a 3.5 ps width laser pulse quasi-resonant (0.5 nm blueshifted) on the LPB. The three panels show the time-space graphs of the amplitude (a), phase (b) and k-space (c) cross cuts with a time step of 0.5 ps. The Look-Up Table (LUT) colour scale in the case of the amplitude chart in (a) is relative to the initial top amplitude, and represents an enhancement factor of 4 (in amplitude) at around 10 ps after the pulse arrival, which corresponds to a factor of 16 in density. The time of the pulse arrival in these time-space charts is $t_0 = 6.5$ ps.

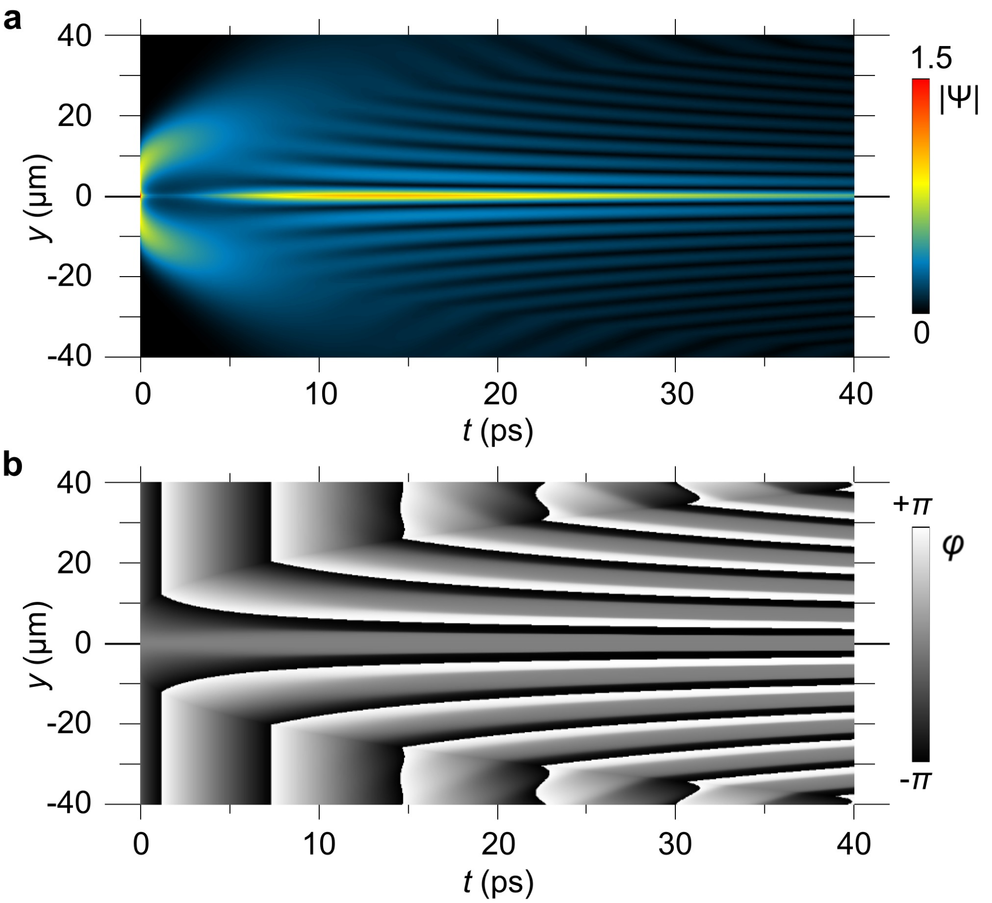

Supplementary Figure 8. Interferometric scenario from a ring source. The ring corresponds to a double gaussian along a diameter cross-cut. The ring radius and width are let to expand in time, which correspond to the two gaussians moving and expanding, respectively. The phase is modulated by a radial in-plane wavevector which slightly increases in time. The part of the ring which reaches the centre emerges on the other side and interferes with the opposing wavevector fluid, causing a structure similar to that observed in the experiment.

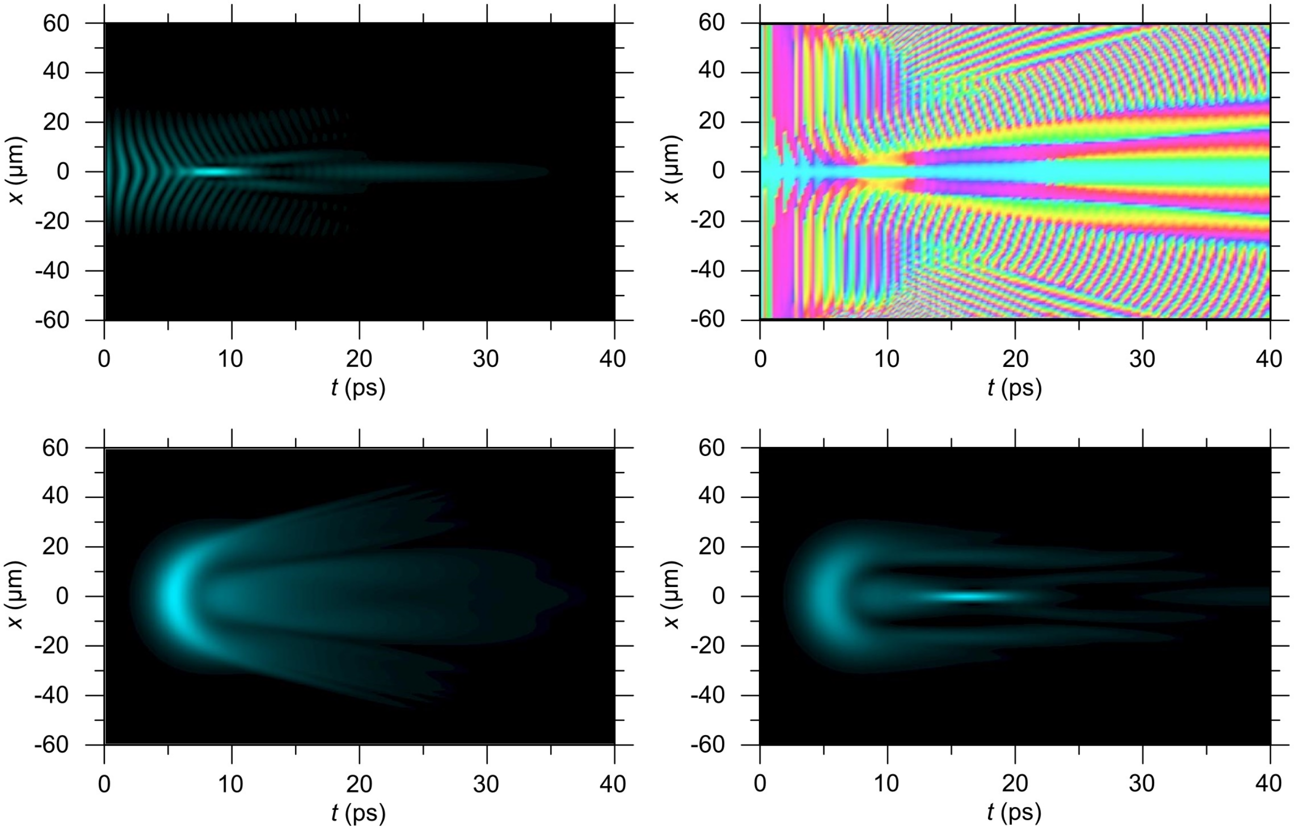

Supplementary Figure 9. Evolution of the photon field intensity in the model with density dependent reduction of the Rabi coupling. Top frames show the photon field density and phase, respectively, in the case of femtosecond pulse excitation. Bottom frames show picosecond pulse excitation at the position of the LP branch (left), failing to produce the localization, and at the position of the UP branch (right), featuring a real-space localization. Parameters are $\Omega_{\rm R} = 2\pi/(0.8\ {\rm ps}) - (3/2\hbar)g|\Psi_{\rm X}|^2$, $\delta = -0.5$ meV, $m_{\rm C} = 3\times10^{-5}\ m_{\rm e}$, $\tau_{\rm C} = 4$ ps, $\tau_{\rm X} = 300$ ps, $\gamma, \eta = 0$, $g = 2\times10^{-2}$ meVμm^2, $\tilde{g} = g$, $\hbar R = 2.6\times10^{-3}$ meVμm^2, $W = 12.5$ μm, $\Delta\omega = 0$, $n_{\rm R} = 0$, $T_{\rm p} = 50$ fs (fs-pulse case) or $T_{\rm p} = 2$ ps (ps-pulse case).

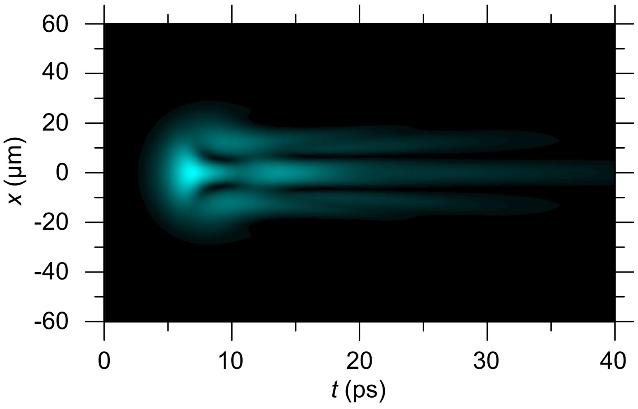

Supplementary Figure 10. Evolution of the photon field intensity in the model with k-dependent nonlocal reduction of the Rabi coupling. Parameters are the same as in Supplementary Figure 9 (for ps pulse excitation at the LP branch) with $a = 4$ and $k_{\rm c} = 0.2$ μm^{-1}.

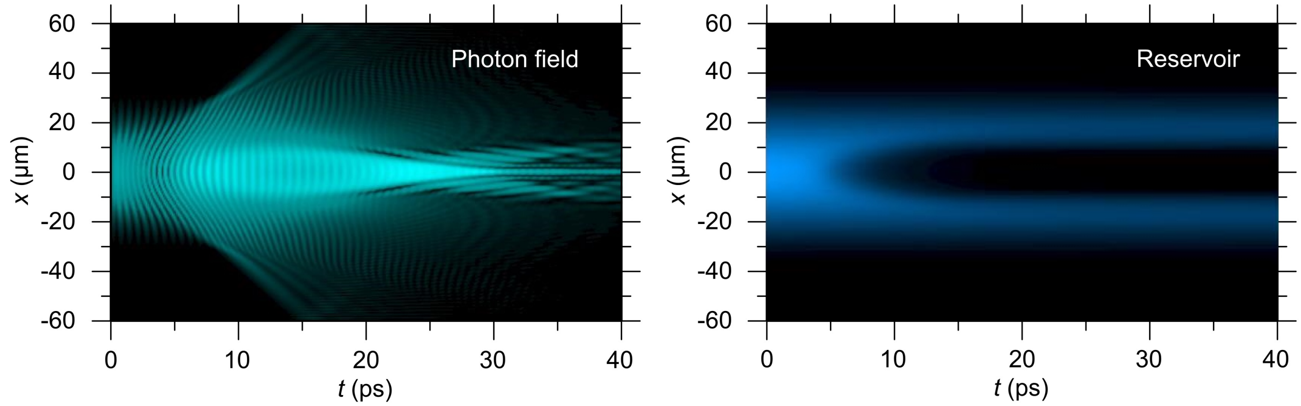

Supplementary Figure 11. Evolution of the photon field intensity and of the reservoir density in the reservoir-ring model. Parameters are $A = 0$, $\delta = 0$, $m_C = 5 \times 10^{-5}\ m_e$, $\hbar\Omega_R = 6$ meV, $\tau_C = 2.5$ ps, $\tau_X = 300$ ps, $g = 12 \times 10^{-3}$ meV μm^2, $\tilde{g} = 2g$, $\hbar R = 2.6 \times 10^{-3}$meVμm^2, $W = 12.5$ μm, $T_p = 50$ fs, $\Delta\omega = 0$.

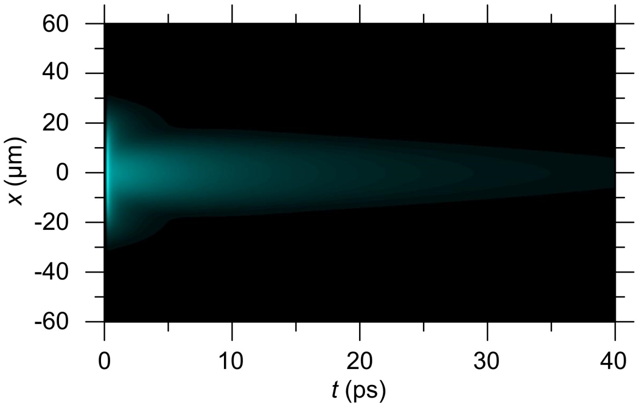

Supplementary Figure 12. Evolution of the photon field intensity in the model with dark excitons. Parameters are the same as in Supplementary Figure 11, except $m_{LP} = 3\times10^{-5}\ m_e$, $\tau_{LP} = 4$ ps, $\gamma = 0.4$ μm^2 ps^{-1}, $\gamma_d = 0.02$ μm^4 ps^{-1}, $g = 2\times10^{-2}$meVμm^2, $n_0 = 0$.

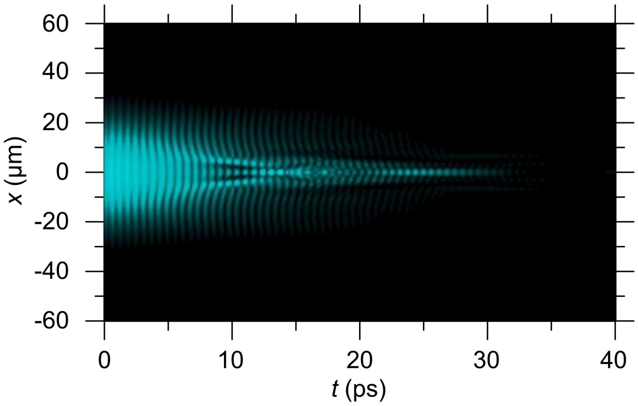

Supplementary Figure 13. Evolution of the photon field intensity in the model with attractive polariton-polariton interactions. Parameters are the same as in Supplementary Figure 11, except $A = 0.1$, $\delta = 3$ meV, $g = -12 \times 10^{-3}$ meVμm^2, $\tilde{g} = 2|g|$, $\hbar R = 4.6 \times 10^{-3}$ meVμm^2.

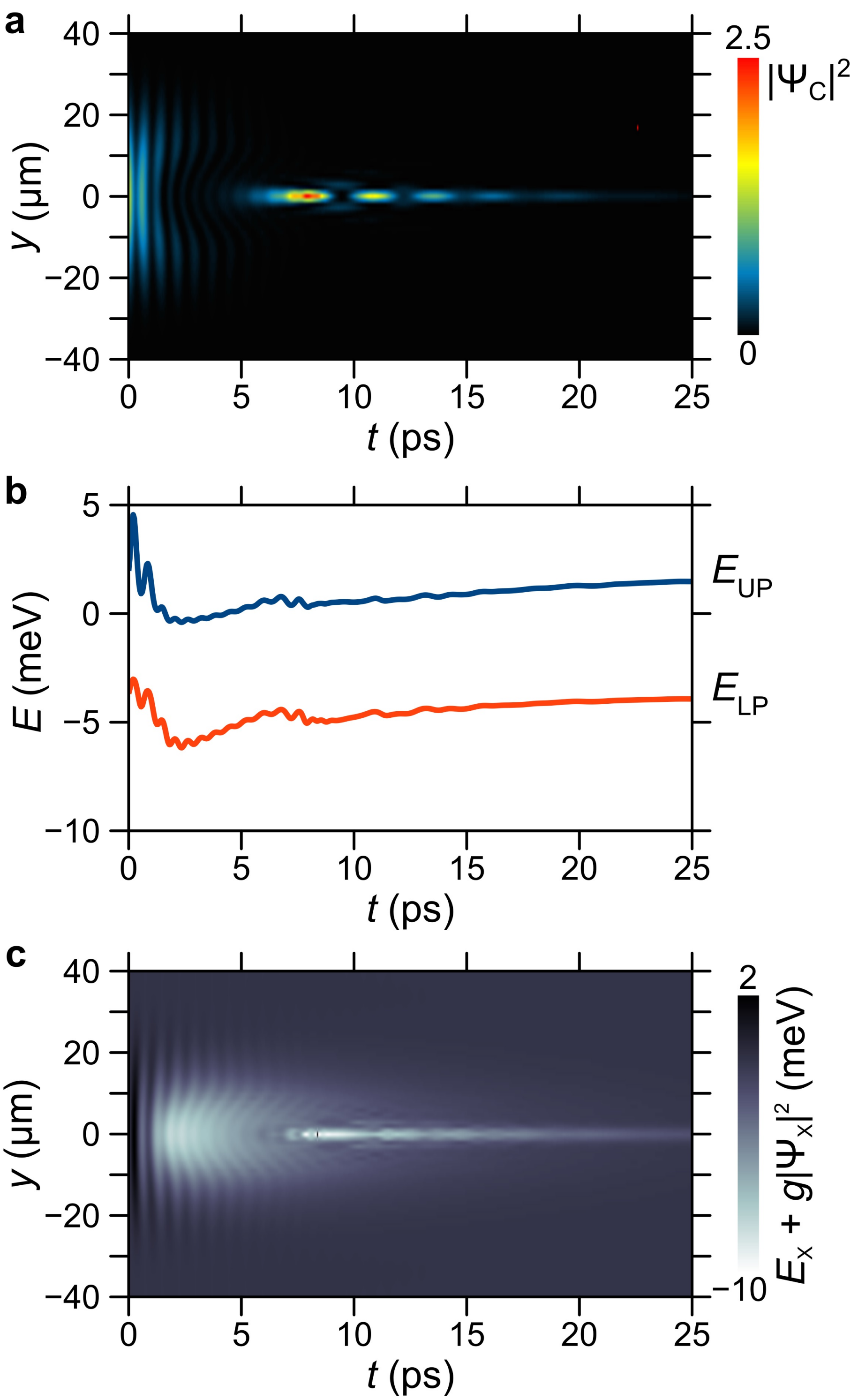

Supplementary Figure 14. Collective polaron model. (a) Time-space chart of the photonic fraction intensity distribution. (b) Time dependence of the local energies of the upper polariton and lower polariton branches. (c) Time-space chart of the effective exciton potential. Parameters used in the calculations are $\Omega_\mathrm{R} = 2\pi/(0.8\ \mathrm{ps})$, $\Delta = -1$ meV, $m_\mathrm{C} = 3 \times 10^{-5}$, $\tau_\mathrm{C} = 4$ ps, $\tau_\mathrm{H} = 8$ ps, $g = 2 \cdot 10^{-2}$ meVµm^2, $\beta = g/n_\mathrm{sat}$, $n_\mathrm{sat} = 3 \times 10^{10}$ cm^{-1}. The pumping field is described by a fs-pulse with $W = 12.5$ µm, $T_\mathrm{p} = 50$ fs, $E_\mathrm{p} = 0$, and a variable amplitude F_p (see text).

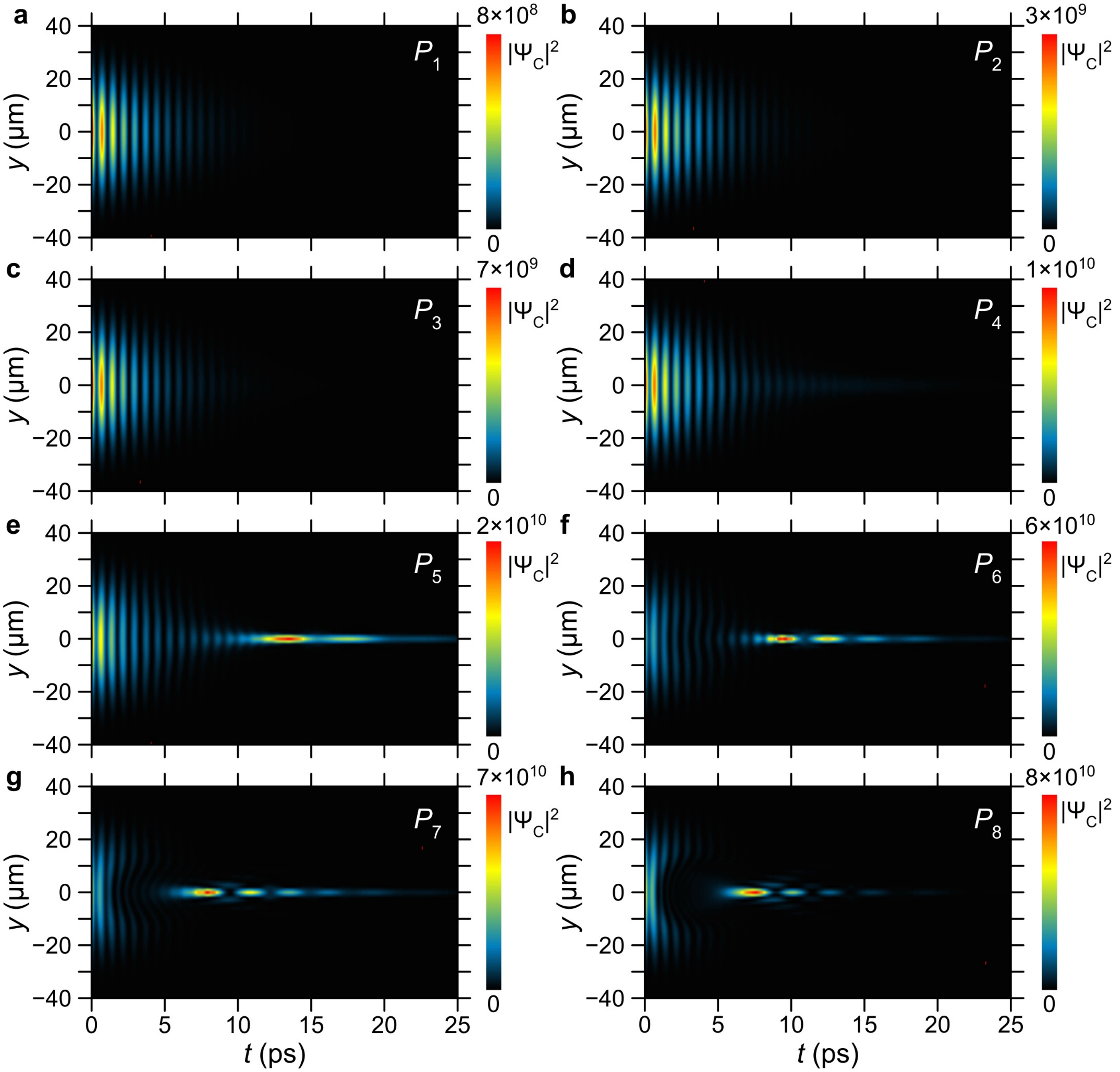

Supplementary Figure 15. Collective polaron model. Calculated time-space charts of the photonic component of the polariton wavefunction as in Supplementary Figure 14a. The number of polaritons is increasing proportionally from P_1 to P_8. The other parameters used in the calculations are listed in caption to Supplementary Figure 14.

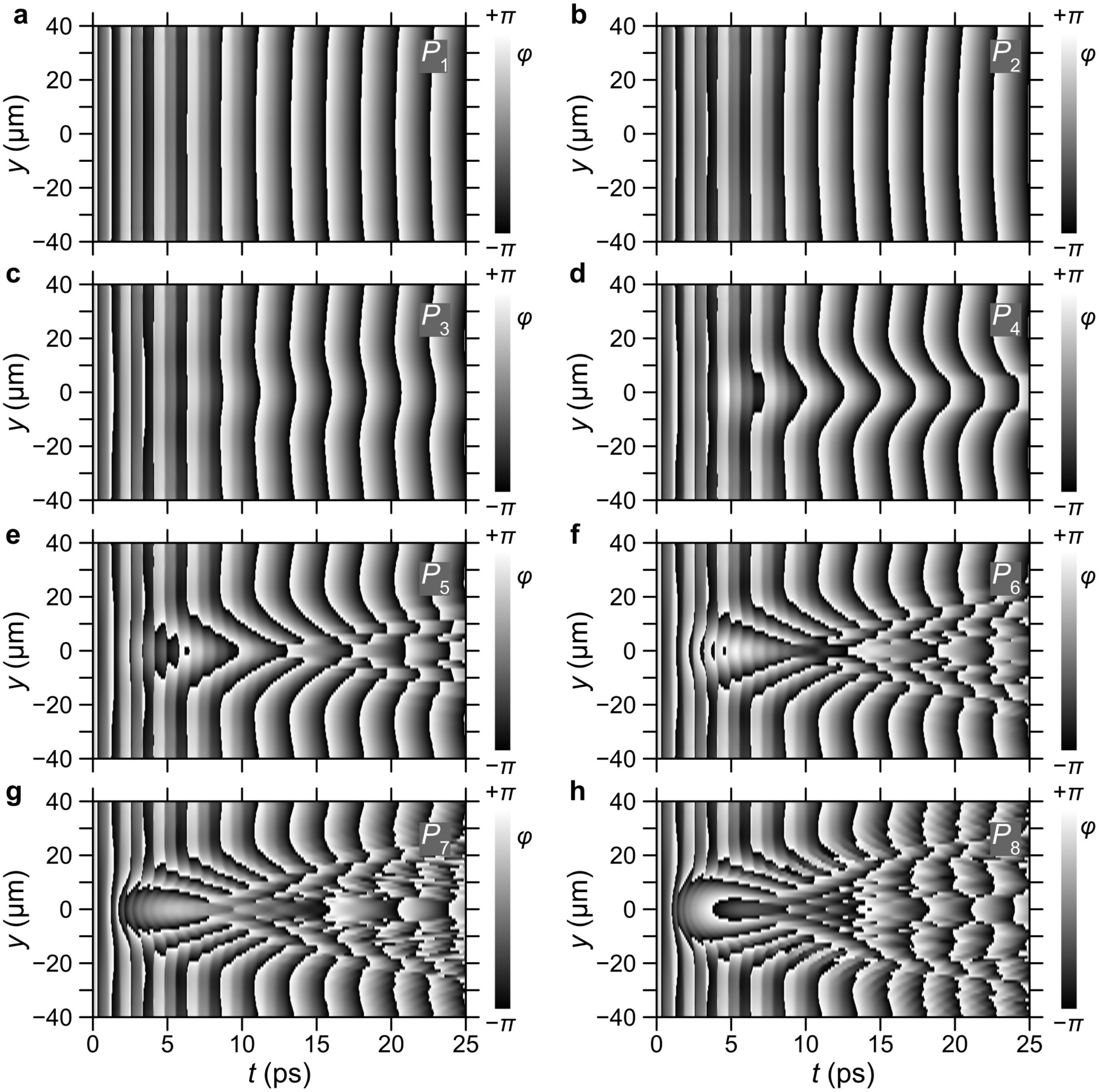

Supplementary Figure 16. Collective polaron model. Calculated time-space chart of the photonic phase at different powers if using a pulsed excitation, as in Supplementary Figure 15.

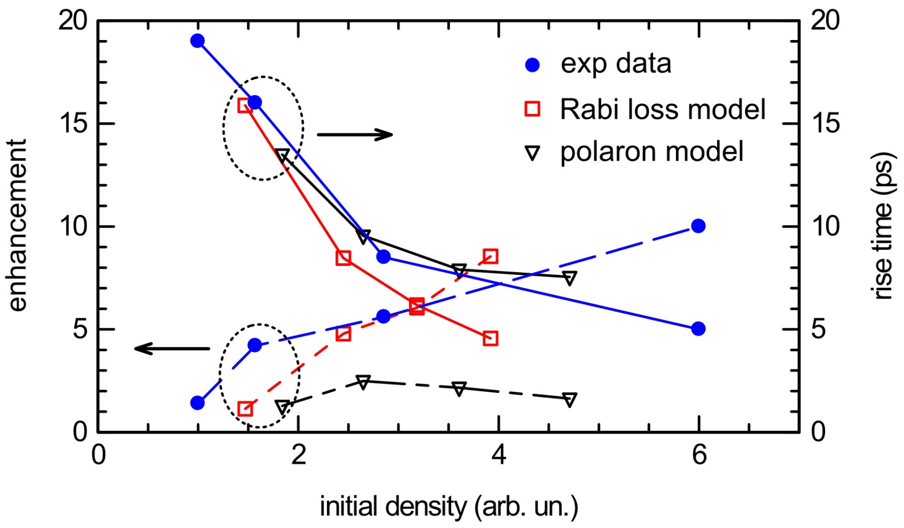

Supplementary Figure 17. Comparison of enhancement factor and rise time between theory and models. The quantitative data have been extracted from the fs experiments series of Supplementary Figure 3 (powers P_3–P_6), the model with loss of Rabi coupling (fs excitation, as in Supplementary Figure 9) and from the collective polaron model of Supplementary Figure 15 (powers P_5–P_8). The respective densities are scaled through the parameter gn. Note that while the Rabi loss model provides a better quantitative agreement, it fails at a qualitative level since it results in attractive attractions only for the upper polaritons and thus cannot explain the observation for the lower polaritons alone.

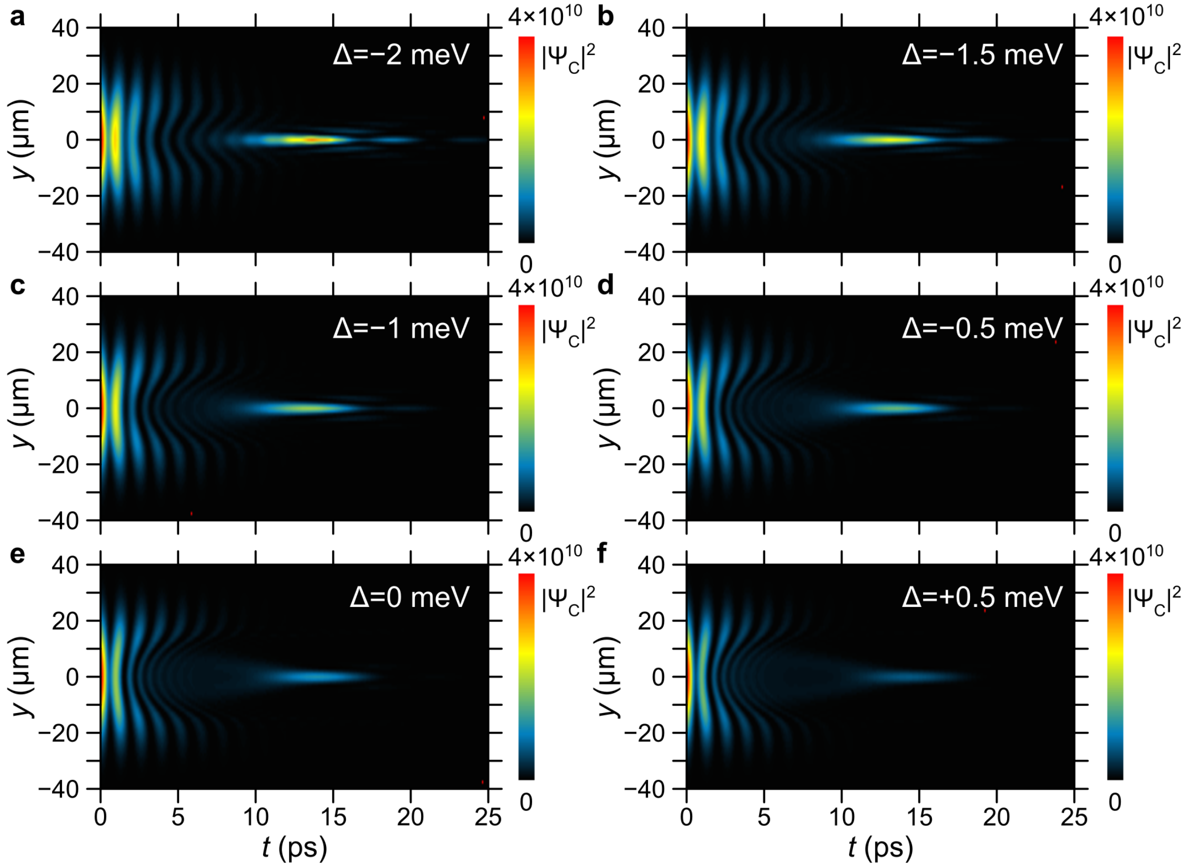

Supplementary Figure 18. Collective polaron model. Calculated time-space chart of the photonic density at different microcavity-exciton detuning if using a pulsed excitation, as in Supplementary Figure 15.

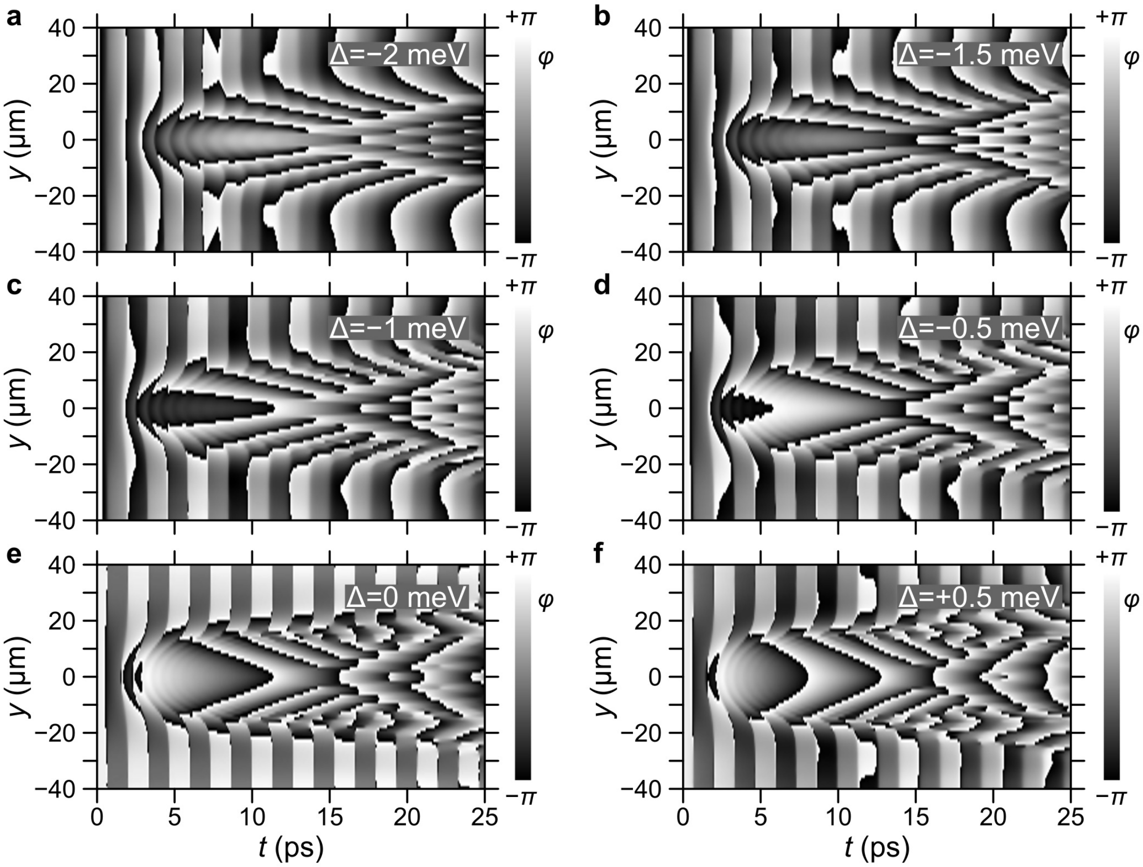

Supplementary Figure 19. Collective polaron model. Calculated time-space chart of the photonic phase at different microcavity-exciton detuning if using a pulsed excitation, as in Supplementary Figure 15.

SUPPLEMENTARY NOTES

Supplementary Note 1. Theoretical models.

The model commonly used to describe the dynamics of polaritons is based on the mean-field approach. The time evolution of the photonic (excitonic) wavefunctions $\Psi_{\mathrm{C}}(\mathbf{r})$ ($\Psi_{\mathrm{X}}(\mathbf{r})$) is given by coupled Gross-Pitaevskii equations [1, 2]:

$$\begin{aligned} i\hbar\frac{\partial\Psi_{\mathrm{C}}}{\partial t} &= (E_{\mathrm{C}} - \frac{\hbar^2}{2m_{\mathrm{C}}}\nabla^2)\Psi_{\mathrm{C}} + \frac{\hbar\Omega_{\mathrm{R}}}{2}\Psi_{\mathrm{X}} - \frac{i\hbar}{2\tau_{\mathrm{C}}}\Psi_{\mathrm{C}} + F_{\mathrm{p}}(\mathbf{r},t), \\ i\hbar\frac{\partial\Psi_{\mathrm{X}}}{\partial t} &= (E_{\mathrm{X}} - \frac{\hbar^2}{2m_{\mathrm{X}}}\nabla^2)\Psi_{\mathrm{X}} + \frac{\hbar\Omega_{\mathrm{R}}}{2}\Psi_{\mathrm{C}} + g|\Psi_{\mathrm{X}}|^2\Psi_{\mathrm{X}}, \end{aligned} \tag{1}$$

where m_{C} (m_{X}) is the cavity photon (exciton) effective mass, g is the exciton-exciton interaction constant, Ω_{R} is the Rabi frequency determining the exciton-photon coupling, $F_{\mathrm{p}}(\mathbf{r},t) = F_0 e^{-\mathbf{r}^2/2W^2 - t^2/2T_{\mathrm{p}}^2 - iE_{\mathrm{p}}t/\hbar}$ is the pumping field describing the pulsed excitation of the cavity with a Gaussian spot of diameter $2W$ and pulse-duration of $2T_{\mathrm{p}}$ and F_0 is a parameter determining the density of photo-created carriers. While this model has been successful to describe much of the fluid dynamics of polaritons, including ballistic motion [3, 4], superfluidity [5], solitons [6], etc. [7], in presence of repulsive interactions, $g > 0$, it cannot account for real-space localization. Many additional ingredients to these equations can produce qualitatively the most important feature of our experiment, the formation of a high density peak in the center. However, these extended models, based on different physical assumptions, have some implications, present other qualitative features and/or require certain ranges or parameters that rule them out as an explanation of the phenomenon. Some hypotheses that work reasonably well demand assumptions that are difficult to justify, such as explicit attractive interactions, $g < 0$. We found that one model only is sufficiently consistent with all the observations and provides a reasonably close agreement with the data to be retained as a possible mechanism for our experiment. It is presented last in a series of alternative descriptions, below: the loss of strong coupling in Supplementary Note 2, a localisation due to the exciton reservoir or dark excitons in Supplementary Note 3, polaritons with attractive interactions in Supplementary Note 4—all of these models failing in some fatal way to account for the experiment—and, ultimately, the collective polaron effect, in Supplementary Note 5, which, on the contrary, reproduces adequately the findings, based on likely assumptions and parameters corresponding to our sample.

Supplementary Note 2. Loss of strong-coupling.

The measured emission of total emitted photons from a single experiment suggests that the exciton density is close to the saturation value of $n_{\mathrm{sat}} \approx 10^3 \mu\mathrm{m}^{-2}$. At such a high density, the reduction of the Rabi splitting should occur due to phase space filling [1, 8–11] and lead to a modification of the polariton dispersion. At the first order of perturbation in the exciton density, the saturation is momentum-independent, or local in space (precisely, the range of the interaction is of the order of the exciton radius) [9–11]. The linear regime Rabi coupling Ω_{R0} is then renormalized to:

$$\Omega_{\mathrm{R}} = \Omega_{\mathrm{R0}} - a\frac{g|\Psi_{\mathrm{X}}|^2}{\hbar} \tag{2}$$

where a is the coefficient dependent on the quantum well geometry, g the polariton-polariton interaction strength and Ψ_{X} the exciton wavefunction (as already defined before). This leads to effective attractive interactions but only for the UP branch, while the interactions remain repulsive for the LP branch [1]. This is because the UP branch is redshifted whilst the Rabi coupling decreases, while the LP branch is blueshifted, just as it would be from repulsive interactions. A numerical simulation for this mechanism is shown in Supplementary Figure 9. This model correctly reproduces the experimental Rabi bending and allows to describe a central density peak with both a femtosecond pulse and a picosecond pulse tuned to the UP branch. Critically, however, the ps pulse excitation tuned to the LP branch leads to strong defocusing in this model, in contradiction with the experimental data.

To overcome the defocusing in the case of LP branch excitation, we can consider a momentum-dependent loss of the Rabi coupling. Assuming a stronger reduction of Ω_{R} in the vicinity of $k = 0$, where lies the condensate whose density is responsible for the phase-space filling, allows to create a negative effective mass for the lower polariton. Indeed, the LP polariton branch blueshifts more at $k = 0$, creating a negative curvature of the dispersion around the ground state, allowing for self-focusing even with repulsive interactions. Technically, this can be achieved by filtering Eq. (2) in k space. In the simplest formulation, this is done by transforming the nonlinear fields $\Psi'_{\mathrm{X}} = |\Psi_{\mathrm{X}}|^2\Psi_{\mathrm{X}}$ and $\Psi'_{\mathrm{C}} = |\Psi_{\mathrm{X}}|^2\Psi_{\mathrm{C}}$ to k-space, applying a Gaussian filter $\Psi'_{\mathrm{X,C}}(k) = \Psi'_{\mathrm{X,C}}(k)\exp(-k^2/2k_c^2)$, transforming them back,

and subtracting from the $(\hbar\Omega_{\rm R}/2)\Psi_{\rm X,C}$ terms in Eqs. (1) after multiplication by $ag/2$. This nonlocal and nonlinear reduction of the Rabi coupling is equivalent to introducing interactions that are nonlocal in real space. The range of the nonlocal effective interaction is given by $k_{\rm c}^{-1}$. Supplementary Figure 10 shows an example of self-focusing in this model in the case of picosecond LP branch excitation. In the case of femtosecond excitation, the results are similar to the ones obtained with the previous model.

While this model provides a good agreement with experiments performed at different settings, its justification is not straightforward. While the k-dependent reduction of Rabi coupling can be predicted by the simple model of phase space filling or exchange effects [8], it occurs at k vectors comparable to the inverse exciton Bohr radius $a_{\rm B}^{-1}$. The dependence of the $\Omega_{\rm R}$ on momentum for k comparable to $\mu{\rm m}^{-1}$ requires an existence of correlations over distances much larger than the exciton radius, for which we see no rationale in the mean-field framework. An explanation along these lines is thus thwarted in a straightforward model and would require a drastic reconsideration of the physics of polaritons based on the mean-field approximation and perturbation theory in the exciton basis. In the close vicinity of the Mott transition, the description in terms of the dilute exciton gas may become questionable, and collective effects of strongly-correlated quantum gases could be required instead. Indeed, BCS-related physics is expected to occur close to the phase space filling threshold [12]. Such an interpretation cannot be excluded and would open a new page of the field, but it is, however, much beyond the scope of this text.

Supplementary Note 3. Localisation by the reservoir.

The exciton reservoir is another possible candidate to explain the redistribution of the polariton condensate. A conceivable scenario involves the creation of a ring of reservoir excitons around the center of the pumping spot, which subsequently creates a backflow of polariton waves, interfering constructively in the center and giving rise to the strong intensity peak. Such a model consists of the photon, exciton, and reservoir fields, similar to Ref. [13], but treating coherent photons and excitons with separate fields:

$$
\begin{aligned}
i\hbar\frac{\partial\Psi_{\rm C}}{\partial t} &= -(1-iA)\frac{\hbar^2}{2m_{\rm C}}\nabla^2\Psi_{\rm C} + \frac{\hbar\Omega_{\rm R}}{2}\Psi_{\rm X} - \frac{i\hbar}{2\tau_{\rm C}}\Psi_{\rm C} + F_{\rm p}(\mathbf{r},t)\,,\\
i\hbar\frac{\partial\Psi_{\rm X}}{\partial t} &= \frac{\hbar\Omega_{\rm R}}{2}\Psi_{\rm C} - i\delta\Psi_{\rm X} + g|\Psi_{\rm X}|^2\Psi_{\rm X} + \tilde{g}n_{\rm R}\Psi_{\rm X} + i\frac{\hbar}{2}\left(Rn_{\rm R} - \frac{1}{\tau_{\rm X}}\right)\Psi_{\rm X}\,, \qquad (3)\\
\frac{\partial n_{\rm R}}{\partial t} &= -\left(\frac{1}{\tau_{\rm X}} + R|\Psi_{\rm X}|^2\right)n_{\rm R}\,,
\end{aligned}
$$

where A is the energy relaxation constant, $F_{\rm p}(\mathbf{r},t) = F_0{\rm e}^{-\mathbf{r}^2/2W^2 - t^2/2T_{\rm p}^2 - i\Delta\omega t}$ is the pumping field and with an initial reservoir density $n_{\rm R}(\mathbf{r},t=0) = n_0{\rm e}^{-\mathbf{r}^2/2W^2}$. In this simple model, the backscattering from the condensate to the reservoir is not taken into account, but the initial reservoir density is assumed to be excited by the incoming pulse.

A numerical simulation of this model is shown in Supplementary Figure 11. The depletion in the center of the reservoir is created due to the stimulated scattering to the condensate, which is faster in the center, where the condensate density is larger. The bright density peak is then created because the polariton waves emitted form the remaining ring-shaped reservoir "focus" in the center. While the main feature of the experiment is thus reproduced, there are several problems with quantitative features: (a) the central peak appears much later than is observed in the experiment and (b) the bending of the Rabi oscillations is initially in the incorrect direction (backwards in the center). There are even more serious conceptual difficulties: (c) in such a model, the peak cannot move "ballistically", as is observed in the experiment if the excitation pulse is injected at an angle, since the reservoir would stay essentially in the position where it is created. (d) The peak persists when resonantly exciting the lower branch, which is expected to produce little or no reservoir excitons. (e) Finally, the effect does not show any strong polarization dependence. In the regime of quasiresonant pulsed excitation we expect the reservoir to be polarised as the typical exciton spin-relaxation time in our system is supposed to be long compared to the characteristic time scale of the Rabi-oscillations and collapse dynamics

Variations on this theme are possible. For instance, one can assume the intermission of dark excitons. Since the coupling is incoherent and assuming the exciton mass as infinite, which allows to treat dark excitons as a density field, their effect can be modeled by the following equations:

$$
\begin{aligned}
i\hbar\frac{\partial\Psi_{\rm LP}}{\partial t} &= \left(-\frac{\hbar^2}{2m_{\rm LP}}\nabla^2 - \frac{i\hbar}{2\tau_{\rm LP}} - i\gamma|\Psi_{\rm LP}|^2 + i\gamma_{\rm d}n_{\rm dark}^2 + g\left(|\Psi_{\rm LP}|^2 + n_{\rm dark}\right)\right)\Psi_{\rm LP} + F(\mathbf{r},t), \qquad (4)\\
\frac{\partial n_{\rm dark}}{\partial t} &= \gamma|\Psi_{\rm LP}|^4 - \gamma_{\rm d}n_{\rm dark}^2|\Psi_{\rm LP}|^2.
\end{aligned}
$$

A numerical simulation of this model is shown in Supplementary Figure 12. The scattering to polaritons creates a hole in the Gaussian distribution of excitons. The flow towards the hole can create a localization, however, the effect is not as clear as in the experiment: the localization is rather broad and has a low intensity.

Supplementary Note 4. Attractive interactions between excitons.

The interaction constant in the Gross-Pitaevskii equations, and variants, that describe the polariton dynamics is positive, as indeed same-spin excitons are largely believed to be repulsive, but several effects could potentially give rise to attractive interactions, as we discuss shortly. Since we actually observe a real-space collapse, it is instructive to simply assume attractive interactions between polaritons. In this case self-focusing is indeed possible for a positive effective mass. The model is identical to the one above, Eqs. (1), but with $g < 0$. We do not include the reservoir excitons that we have seen can be ruled out on several grounds. A result of numerical simulation is shown in Supplementary Figure 13. As expected, the high intensity peak appears due to self-focusing.

While some features of the experiment are well reproduced in this model, the existence of attractive polariton interactions is highly questionable. Potentially the strongest effect in this sense is the indirect exchange through the biexciton state, but this occurs only for opposite spins, while we did not observe any strong spin dependence of the focusing effect in the experiment. The other possible sources of attractive interaction, such as the indirect exchange [14–16], or Van der Waals interactions appear to be negligible for the low-momentum excitons created by resonant pumping [16]. It was also recently observed that the sign of the interactions may change to negative at large polariton momenta [15], and other effects could similarly give rise to polariton attraction [15, 16], but the proposed configurations are difficult to fit in with our experiment. The change of sign of the polariton interactions with no further justification is thus not tenable and would be in conflict with a large literature that successfully describes various experimental regimes on a physically well motivated assumption of repulsive interactions.

Supplementary Note 5. Collective polaron effect.

We now conclude this series of possible models to reproduce the real-space collapse of the polariton condensate with the one model that we found could stand as an explanation for the observed effect.

When the exciton density is large enough, as in the case of pulsed excitation, the macroscopic occupation of the ground state with excitons may lead to enhancement of the exciton-phonon interaction that produces a cooling of the excitonic reservoir against its blueshift due to their attraction [17, 18]. The corresponding released energy is transfered to the crystal lattice via acoustic phonons. Another plausible mechanism of heating is through the polariton-polariton Auger process followed by emission of the cascade of acoustic phonons as discussed by Klembt *et al.* [19]. In any case, a local heating may occur which leads to a band-gap narrowing, a variation of the exciton-photon detuning and a redshift of the lower polariton branch. The latter effect may lead to self-focusing of the polariton condensate. Such an effect can be referred to as a "collective polaron effect". Formally, it can be described by the modulation of the exciton energy through a retarded function:

$$E_{\mathrm{X}}(t) = -\frac{\beta}{\tau_{\mathrm{H}}} \int_{-\infty}^{t} \left|\Psi_{\mathrm{X}}\left(\mathbf{r}, t'\right)\right|^{4} e^{-\frac{t-t'}{\tau_{\mathrm{H}}}} dt', \tag{5}$$

where β characterises the magnitude of band-gap renormalization and τ_{H} is the characteristic heat relaxation time in the crystal lattice. Both β and τ_{H} are fitting parameters. The fourth power of the exciton wavefunction $\Psi_{\mathrm{X}}(\mathbf{r}, t')$ under the integral accounts for the quadratic dependence of the polariton Auger process on the concentration of polaritons. The dynamics of the system is governed by the initial pumping strength. The Rabi oscillations generally have an anharmonic behavior due to the changes of the bare exciton energy and the interplay between the blueshift $g|\Psi_{\mathrm{X}}|^2$ and the redshift $E_{\mathrm{X}}(t)$. The effective frequency of these oscillations is then determined by $\Omega_{\mathrm{eff}} = \sqrt{\Omega_{\mathrm{R}}^2 + (\Delta - g|\Psi_{\mathrm{X}}|^2)^2}$ [20]. In addition to the change in detuning, at larger population densities close to the Mott transition, the coupling Ω_{R} itself would be reduced, resulting from the change of the exciton oscillator strength due to the exciton phase space filling [8].

The result of the numerical simulation of Eqs. (1) with an Eq. (5) dependence is shown in Supplementary Figure 14. At the initial stage, the Rabi oscillations between photon and exciton states are seen. The dependence of the oscillations frequency on the local detuning results in appearance of the radial waves spreading from the center of the spot. The modified density-dependent value of the Rabi frequency allows to reproduce some retardation of their

motion in the spot center. When the density decreases due to the photonic decay, the direction of the wave-spreading changes. In addition, the heating reservoir forms a potential well, in which the polaritons are trapped through their excitonic fraction, that results in a stabilization of both excitonic and photonic components of the polariton flow. Further dynamics exibits the decay of both components of the polariton many-body wavefunction. One can see the typical behavior of the sharply localised peak appearance extracted at different pumping power, which is modeled by setting increasing amplitude of F_{p} in Eq. (1). The results are shown in Supplementary Figure 15. The photonic phase component is plotted in Supplementary Figure 16.

A comparison of the quantitative values for the enhancement factor and rise time, between the experiments, the polaron model and the Rabi loss model, are presented in the plot of Supplementary Figure 17. The model with loss of coupling shows a better agreement for the enhancement factor but we recall that it was working only in the fs case for its action on the UPB, and not with the exclusive excitation of the LPB. The agreement with experiments for the polaron model is showing some deviations in the enhancement factor at the larger density, while remaining very good for the rise time values. Finally, we report the behavior of the polaron model at different microcavity-exciton detuning, by plotting the photonic density and phase, Supplementary Figure 18 and Supplementary Figure 19, respectively. This shows that while the rise time remains similar (i.e., mainly depending on the pump power), the enhancement factor within the polaron model is larger at negative microcavity-exciton detuning.

Our collective polaron model yields the best fit to the data with a heat relaxation time $\tau_{\mathrm{H}} = 8$ ps (see Eq. (5) and caption to Supplementary Figure 14). This seems to be a correct order of magnitude, as one can see from the comparison with the thermal relaxation time in GaAs calculated by Luo *et al.* for different scattering mechanisms [21], in the low temperature limit. The thermal conductivity of GaAs is estimated by Luo *et al.* as 10 W mK^{-1} at the low temperature limit. The temperature gradient of about 5 K μm^{-1} would induce an energy flow of 0.05 mW. With a threshold of polariton lasing of the order of 10 mW in GaAs based microcavities, this seems to be a correct order of magnitude. The fluid redistribution dynamics and the heat relaxation dynamics appear to be of the similar timescales. A detailed information on cooling/heating of the crystal lattice in the presence of exciton-polaritons is given in Klembt *et al.* [19]. From the comparison with the paper by Luo, it follows that about 5% of the energy optically injected to the microcavity is likely to be thermally dissipated, the rest being radiatively emitted (with radiative lifetime, say, 10 ps). If the thermal dissipation rate is 20 times lower, we end up with the effective heat wave which would propagate over a distance comparable with the spot size (20 μm) in 20·10 ps = 200 ps. This yields the speed of 0.1 μm ps^{-1}. For comparison, in another work, the actual propagation rate of the heat wave is determined by the acoustic sound velocity, which is of the order of 0.4 μm ps^{-1} for GaAs in Matsuda *et al.* [22]. Note that in reality there is no single heat wave but rather the heat propagates diffusively.

In the present model as well, we assume that the incoherent reservoir is either completely empty or does not play any role. This assumption is certainly valid in the case of selective picosecond excitation of the lower polariton (LP) branch. We note also that the weak-coupling saturable optical nonlinearity could arise from instantaneous effects or sample heating [23]. The instantaneous part, however, provides only defocusing nonlinearity for excitation below the band edge, as follows from the nonlinear Kramers-Kronig relations [23, 24]. Heating generally leads to redshift which corresponds to focusing nonlinearity but has a longer response time.

Supplementary Note 6. Other Mechanisms.

It is not excluded that more exotic many-body physics is at play, such as a variation of the BCS mechanism, holding the Bosonic condensate together in a way similar as the superconducting phase molds the Fermion liquid, or a dynamical Casimir effect [25, 26] pulling non-zero-momentum particles out of the suddenly hit polariton vacuum. This last interpretation deserves a special mention as it is so closely related to our experiment. If someone would want to check the dynamical Casimir effect as proposed in the works just cited, they would prepare an experiment close to that described in our work. The theory, based on the Truncated Wigner Approximation (TWA), is however yet not ripe to be confronted to our observations. It has indeed so far be implemented only in 1D (though for both homogeneous [25] and inhomogeneous [26] systems). Dimensionality could be crucial in our case. Nevertheless, a preliminary comparison could be that, as stated in these works, the “zero pulse width approximation” (see Ref. [25] Eq. 2) holds for a pulse duration $\tau_{\mathrm{L}} < \hbar/[gn_{\mathrm{c}}(0)]$, with $gn_{\mathrm{c}}(0)$ the initial blueshift of the condensate. With blueshifts of 0.5 meV, 1.0 meV or 1.5 meV, this means $\tau_{\mathrm{L}} < 1.3$ ps, 0.65 ps and 0.43 ps, respectively. This holds in the case of the fs (130 fs) experiments we show, while the ps (3.5 ps) experiment is at the border of this range (when imparting a blueshift less than 0.2 meV). If the pulse duration is less than any other time scale of the dynamics, it only sets the initial condition of the polariton gas which then can freely evolve. Another quantity to consider is the amount of finite-k particles generated. In the 1D homogeneous case, this reaches a 10% or 20% fraction of the k=0 condensate, starting from an initial blueshift of 0.5 meV and 1.5 meV, respectively (see [25] Fig. 3), supposedly enough to produce a visible structure. For the inhomogeneous case (Gaussian), the finite-k state is ejected initially with a velocity which

then decreases, similarly to the experiments, though it reaches lower values (0.25 μm^{-1}) than in the experiment (1.25 μm^{-1}). The ring in k-space is however ascribed to the repulsive expansion of the condensate and there is no evidence of a ring in real space. In any case, these magnitudes, as well as their manifestation depend strongly on the dimensionality of the system, and in a 2D polar system the same amounts of particles could give rise to very different phenomenology (we recall that just $\sim$6% of the particles contribute to the bright peak of the experiments). At this stage any further consideration is just speculation and any quantitative comparison could be misleading, without the implementation of the 2D model. While the collective polaron model provides a better agreement with the observations, we should therefore exert caution at this stage not to discard alternative beyond mean-field effects, that could provide further insights into the dynamics at play.

SUPPLEMENTARY DISCUSSION

In conclusions, while there is no compelling argument that impose the collective polaron effect as the mechanism causing the collapse of the polariton condensate, our simulations show that it is a reasonable candidate, when other more straightforward scenarios fail to provide even a qualitative agreement or demand assumptions too strong to be justified. We feel that it is left to theorists and further investigations to elucidate which exact physics is causing the peculiar phenomenology that we report. In any case, involving self-localization by the phonon field as suggested by our model, or through the manifestation of a more complex, as yet unidentified, many-body mechanism, our observations establish a rich, unsuspected and potentially useful dynamics of ultra-fast and ultra-dense bosonic gases, as well as the predispositions of polaritons for the study of such regimes.

SUPPLEMENTARY REFERENCES

1. Ciuti, C., Schwendimann, P. & Quattropani, A. Theory of polariton parametric interactions in semiconductor microcavities. *Semicond. Sci. Technol.* **18**, S279–S293 (2003).
2. Carusotto, I. & Ciuti, C. Probing microcavity polariton superfluidity through resonant rayleigh scattering. *Phys. Rev. Lett.* **93**, 166401 (2004).
3. Steger, M. *et al.* Long-range ballistic motion and coherent flow of long-lifetime polaritons. *Phys. Rev. B* **88**, 235314 (2013).
4. Amo, A. *et al.* Collective fluid dynamics of a polariton condensate in a semiconductor microcavity. *Nature* **457**, 291–5 (2009).
5. Amo, A. *et al.* Superfluidity of polaritons in semiconductor microcavities. *Nat. Phys.* **5**, 805–810 (2009).
6. Sich, M. *et al.* Observation of bright polariton solitons in a semiconductor microcavity. *Nat. Photonics* **6**, 50–55 (2012).
7. Carusotto, I. & Ciuti, C. Quantum fluids of light. *Rev. Mod. Phys.* **85**, 299–366 (2013).
8. Schmitt-Rink, S., Chemla, D. S. & Miller, D. A. B. Theory of transient excitonic optical nonlinearities in semiconductor quantum-well structures. *Phys. Rev. B* **32**, 6601–6609 (1985).
9. Rochat, G. *et al.* Excitonic bloch equations for a two-dimensional system of interacting excitons. *Phys. Rev. B* **61**, 13856–13862 (2000).
10. Kwong, N. H., Takayama, R., Rumyantsev, I., Kuwata-Gonokami, M. & Binder, R. Third-order exciton-correlation and nonlinear cavity-polariton effects in semiconductor microcavities. *Phys. Rev. B* **64**, 045316 (2001).
11. Luk, M. H. *et al.* Transverse optical instability patterns in semiconductor microcavities: Polariton scattering and low-intensity all-optical switching. *Phys. Rev. B* **87**, 205307 (2013).
12. Keeling, J., Eastham, P. R., Szymanska, M. H. & Littlewood, P. B. Bcs-bec crossover in a system of microcavity polaritons. *Phys. Rev. B* **72**, 115320 (2005).
13. Wouters, M. & Carusotto, I. Excitations in a nonequilibrium bose-einstein condensate of exciton polaritons. *Phys. Rev. Lett.* **99**, 140402 (2007).
14. Ciuti, C., Savona, V., Piermarocchi, C., Quattropani, A. & Schwendimann, P. Role of the exchange of carriers in elastic exciton-exciton scattering in quantum wells. *Phys. Rev. B* **58**, 7926–7933 (1998).
15. Vishnevsky, D. V. & Laussy, F. Effective attractive polariton-polariton interaction mediated by an exciton reservoir. *Phys. Rev. B* **90**, 035413 (2014).
16. Vladimirova, M. *et al.* Polariton-polariton interaction constants in microcavities. *Phys. Rev. B* **82**, 075301 (2010).
17. Ivanov, A. L., Littlewood, P. B. & Haug, H. Bose-einstein statistics in thermalization and photoluminescence of quantum-well excitons. *Phys. Rev. B* **59**, 5032–5048 (1999).

18. Porras, D., Ciuti, C., Baumberg, J. J. & Tejedor, C. Polariton dynamics and bose-einstein condensation in semiconductor microcavities. *Phys. Rev. B* **66**, 085304 (2002).
19. Klembt, S. *et al.* Exciton-polariton gas as a nonequilibrium coolant. *Phys. Rev. Lett.* **114**, 186403 (2015).
20. Voronova, N., Elistratov, A. & Lozovik, Y. Detuning-controlled internal oscillations in an exciton-polariton condensate. *Preprint at http://arxiv.org/abs/1503.04231* (2015).
21. Luo, T., Garg, J., Shiomi, J., Esfarjani, K. & Chen, G. Gallium arsenide thermal conductivity and optical phonon relaxation times from first-principles calculations. *Europhys. Lett.* **101**, 16001 (2013).
22. Matsuda, O., Tachizaki, T., Fukui, T., Baumberg, J. J. & Wright, O. B. Acoustic phonon generation and detection in $GaAsAl_{0.3}Ga_{0.7}As$ quantum wells with picosecond laser pulses. *Phys. Rev. B* **71**, 115330 (2005).
23. Taranenko, V., Slekys, G. & Weiss, C. Spatial resonator solitons. In Akhmediev, N. & Ankiewicz, A. (eds.) *Dissipative Solitons*, vol. 661 of *Lecture Notes in Phys.*, 131–160 (Springer Berlin Heidelberg, 2005).
24. Kost, A. Resonant optical nonlinearities in semiconductors. In Garmire, E. & Kost, A. (eds.) *Nonlinear Optics in Semiconductors I*, vol. 58 of *Semiconductors and Semimetals*, 1–53 (Academic Press, 1998).
25. Koghee, S. & Wouters, M. Dynamical casimir emission from polariton condensates. *Phys. Rev. Lett.* **112**, 036406 (2014).
26. Koghee, S. & Wouters, M. Dynamical quantum depletion in polariton condensates. *Preprint at http://arxiv.org/abs/1507.02175* (2015).
